# Characterization of necrosis-inducing NLP proteins in *Phytophthora capsici*

**DOI:** 10.1186/1471-2229-14-126

**Published:** 2014-05-08

**Authors:** Bao-Zhen Feng, Xiao-Ping Zhu, Li Fu, Rong-Fei Lv, Dylan Storey, Paul Tooley, Xiu-Guo Zhang

**Affiliations:** 1 Department of Plant Pathology, Shandong Agricultural University, 61, Daizong Street, Tai’an, Shandong 271018, China; 2 University of Tennessee, Genome Sciences and Technology, Knoxville, TN, USA; 3 Foreign Disease-Weed Science Research Unit, USDA, ARS, 1301 Ditto Ave., Ft. Detrick, MD 21702-5023, USA

**Keywords:** *Phytophthora capsici*, Necrosis-inducing proteins (NLPs), *PcNLP* genes, Chlorotic or necrotic response, mRNA expression, PcNLPs protein expression

## Abstract

**Background:**

Effector proteins function not only as toxins to induce plant cell death, but also enable pathogens to suppress or evade plant defense responses. NLP-like proteins are considered to be effector proteins, and they have been isolated from bacteria, fungi, and oomycete plant pathogens. There is increasing evidence that NLPs have the ability to induce cell death and ethylene accumulation in plants.

**Results:**

We evaluated the expression patterns of 11 targeted *PcNLP* genes by qRT-PCR at different time points after infection by *P. capsici*. Several *PcNLP* genes were strongly expressed at the early stages in the infection process, but the expression of other *PcNLP* genes gradually increased to a maximum at late stages of infection. The genes *PcNLP*2, *PcNLP*6 and *PcNLP*14 showed the highest expression levels during infection by *P. capsici*. The necrosis-inducing activity of all targeted *PcNLP* genes was evaluated using heterologous expression by PVX agroinfection of *Capsicum annuum* and *Nicotiana benthamiana* and by Western blot analysis. The members of the *PcNLP* family can induce chlorosis or necrosis during infection of pepper and tobacco leaves, but the chlorotic or necrotic response caused by *PcNLP* genes was stronger in pepper leaves than in tobacco leaves. Moreover, *PcNLP*2, *PcNLP*6, and *PcNL*P14 caused the largest chlorotic or necrotic areas in both host plants, indicating that these three genes contribute to strong virulence during infection by *P. capsici*. This was confirmed through functional evaluation of their silenced transformants. In addition, we further verified that four conserved residues are putatively active sites in *PcNLP*1 by site-directed mutagenesis.

**Conclusions:**

Each targeted *PcNLP* gene affects cells or tissues differently depending upon the stage of infection. Most *PcNLP* genes could trigger necrotic or chlorotic responses when expressed in the host *C. annuum* and the non-host *N. benthamiana*. Individual *PcNLP* genes have different phytotoxic effects, and *PcNLP*2, *PcNLP*6, and *PcNLP*14 may play important roles in symptom development and may be crucial for virulence, necrosis-inducing activity, or cell death during infection by *P. capsici*.

## Background

Plant cells respond to attack signals from pathogens that activate their systemic defense systems [1]. Pathogens secrete a diverse effector proteins into the apoplast and cytoplasm of host cells. Effector proteins not only function directly as toxins to induce plant cell death, but also suppress or evade plant defense responses, thereby favoring early pathogen colonization [2-7]. While some bacteria and fungi produce structurally diverse cytolytic toxins that kill plant cells directly [8], a much broader group of organisms, including prokaryotes [9-13], and eukaryotic oomycetes (Kingdom Stramenopila) [14-21] and fungi produce necrosis-inducing proteins (NLPs) that cause cell death while stimulating the plant’s immune reaction [22-27]. NLPs were first purified from culture filtrate of *Fusarium oxysporum* f. sp. *erythroxyli* and named ‘necrosis and ethylene-inducing proteins’ (NEP1) [22]. Many other NLPs have been isolated from bacteria, fungi, and oomycete plant-pathogens and there is increasing evidence that the different NLPs have the ability to induce cell death and ethylene accumulation in plants [28,29]. The NLP proteins usually possess an N-terminal secretion signal peptide and are apoplastic effectors that compose a superfamily of secreted phytotoxic proteins [28]. Notably, NLPs are expressed inside cells, which may make them less active, but cell lysis and subsequent release of the proteins into the apoplast induces cell death for some of the constructs [29]. In addition to plasma membrane targets, the association of NLP proteins with nuclei of sensitive plant cells has also been recorded [30]. Most identified NLPs not only trigger cell death but also elicit strong immune responses in a large number of dicotyledonous plants and are frequently associated with plant perception of pathogen-associated molecular patterns (PAMPs) [15,18,25,30].

The disruption of some NLP genes in some pathogens such as *F. oxysporum* f. sp. *erythroxyli* and *Mycosphaerella graminicola* does not reduce their virulence [31,32]. Similarly, mutants of *Bcnep*1 or *Bcnep*2 in pathogenic strains of *Botrytis cinerea* result in virulence similar to that of the wild type strains [33]. However, there is strong evidence that NLPs function as virulence factors that accelerate disease and pathogen growth in host plants. For example, the disruption of both *EccNLP* and *EcaNLP* isolated from *Erwinia carotovora* subsp. *carotovora* and subsp. *atroseptica* result in decreased virulence on potato [12,13]. Likewise, the over-expression of *Nep*1 in a hypovirulent strain of the fungus *Colletotrichum coccodes* markedly increased its virulence toward *Abutilon theophrasti* and extended the host range of this pathogen [34]. NLPPya was identified from *Pythium aphanidermatum*, a species that causes similar responses in host and nonhost dicotyledonous plants [15]. All those reports indicate that NLPs from different pathogens play distinct roles and that the characteristics of NLPs during infection of plants by pathogens merit further exploration.

The genus *Phytophthora* comprises a group of filamentous fungus-like organisms that includes some of the most notorious plant pathogens [18]. Pathogenesis by *Phytophthora* species requires their ability to induce cell death in their hosts [35,36]. Until now, only a few *Phytophthora* NLP proteins have been studied in any detail. PsNLP1 codes for a necrosis inducing protein that is secreted by *P. sojae* during infection of *Nicotiana benthamiana*[18], but the varying patterns of expression of other members of the PsNLP family suggest that it has been a positive selection for diversification of function of genes within the family during infection of soybean [21]. *NPP*1 from *P. parasitica* induces a rapid immune response and mitogen-activated protein kinase activation in its hosts [17,25]. Notably, the NPP gene family of *P. infestans* was shown recently to encode a different type of phytotoxic protein that was not correlated with the sequence of NLPs [37]. The genes *PiNPP*1.1, *PiNPP*1.2, and *PiNPP*1.3 (*Pi* = *P. infestans*) were shown to undergo a diversifying selection in late blight during infection of potato by *P. infestans*[37]. These *PiNPP* genes are similar to *PiNPP*1.1, but not *PiNPP*1.2 or *PiNPP*1.3 encoded the putative secreted proteins that triggered cell death in potato [38]. One *NLPp* gene was identified from *P. parasitica* that induced similar responses in host and nonhost dicotyledonous plants [15]. However, some NLP genes from *P. infestans* and *P. megakarya* were always strongly expressed during the early biotrophic infection phase [19,35]. All these reports suggest that NLPs from *Phytophthora* species have different functions in the infection process, but there has been little done to functionally characterize these proteins. Moreover, expansion of *NLP* gene families in the genomes of *P. capsici*, *P. infestans*, *P. ramorum*, and *P. sojae*, which provided sufficient data for further functional evaluation of relaxed selection by a different process.

The structure of NLPs is remarkably conserved over extraordinary phylogenetic distance. The structure of NLPs of stramenopiles *P. parasitica* and *P. aphanidermatum,* and the bacterium *Pectobacterium carotovorum* have a high level of conservation of a central hepta-peptide motif “GHRHDWE”, and four amino acid residues within their crystallized structures (D93A, H101A, D104A, and E106A) correlate with the qualitative and quantitative biological activities of respective NLPs [39]. The folding of NLPs is also similar to that of cytolytic toxins secreted from marine organisms. Despite the recognized influence of NLPs in the complex plant/pathogen interaction, questions persist concerning NLPs [39]. Are NLPs from unrelated organisms functionally conserved as well? Do the necrotic-inducing activities of NLPs facilitate the pathogen’s ability to infect and induce symptoms? Are the toxic/necrotic and defense-stimulating activities of NLPs mechanistically linked?

*P. capsici* causes various disease symptoms in a number of important vegetable [40] and has been found around world [40-42]. *P. capsici* was originally considered to be specific to pepper, but is now known to cause blight disease on many other plants [43,44]. *P. capsici* also secretes a class of effectors, termed RXLRs, that enable parasitic infection and reproduction during infection of different plants [2,3,45,46]. Secretion and translocation of the effectors require the presence of a signal peptide, followed by a conserved N-terminal RXLR motif [45,47,48]. More than 400 putative candidate RXLR effectors in the *P. capsici* genome have been identified by genome-wide searches for RXLR coding genes [49]. However, the roles of the RXLR effectors in *P. capsici*-host interactions are unknown. The potential studies will reveal the exact roles of RXLR effectors during *P. capsici*–host interactions. Another class of cytoplasmic effectors has been identified in the secreted proteins of *P. infestans*; these cause ‘crinkling and necrosis’ phenotypes, named ‘CRN’ [50], in leaves. CRN proteins share a highly conserved LQLFLAK motif required for translocation and a conserved N-terminal region, and in some cases they have a predictable signal peptide. Approximately 80 full-length CRN coding genes and more than 200 pseudogenes have been identified in the *P. capsici* genome by computational surveys [49]. Feng et al. [51] identified additional secreted proteins of 18 PcNLPs in *P. capsici* as possible virulence factors. Considering the activity of PcNLPs in the induction of cell death, these PcNLPs were proposed to contribute to the transition from biotrophy to necrotrophy [51], in which 11 *PcNLP* genes were shown to be highly expressed during infection by *P. capsici*. However, their functional roles in virulence remain to be determined. Thus, further functional investigation of the PcNLPs should illuminate their roles in the virulence of *P. capsici*. Notably, *INF*1 elicitin induced necrosis activity is required for full virulence of *P. infestans*, *P. sojae,* and *P. cryptogea*[18,38,52-56]. Additionally, several bacterial and fungal pathogens produce elicitins that induce avirulence toward a resistant host species [9-11,14,16,23,53]. At the same time, the function of *INF*1 elicitin has been confirmed to act as an avirulence factor in *P. parasitica*-tobacco interactions [53-55] and has also been proposed to be a component of nonhost resistance of *Nicotiana* species to *P. infestans* and other elicitin-producing *Phytophthora* species [53-55]. Overall, *INF*1 could be regarded as a reference function gene when analyzing the function of NLPs from *Phytophthora* species that secrete a different type of phototoxic protein.

In the current publication we provide an analysis of the function of the 11 highly expressed *PcNLP* genes that have been previously identified in *P. capsici* in our laboratory [51]. Our objectives were to define variation in their function, to use leaf infiltration assays to determine whether any of them play important roles in necrosis or cell death-inducing activity, and to determine whether any of them have phytotoxic activity in host and non host species.

## Results

### Expression patterns of *PcNLP* genes during *P. capsici* infection

The *PcNLP* genes were identified in the *P. capsici* genome on the conserved GHRHDWE motif in the DOE Joint Genome Institute database (JGI: http://genome.jgi.doe.gov/PhycaF7/PhycaF7.download.html) using a TBLASTN program by an expected (E) cut off value <10^-15^. We identified 42 putative NLPs containing the conserved GHRHDWE motif. Among these NPPs, 14 were single copy, while the rest were multicopies ranging from 2–12 [51]. We previously cloned 18 NLPs in *P. capsici* SD33, and named them sequentially from *PcNLP*1 to *PcNLP*18 [51]. Moreover, we then identified 60 putative NLPs in the *P. capsici* genome (JGI: http://genome.jgi.doe.gov/Phyca11/Phyca11.download.html) under these conditions. As shown in Table 1, a total of 15 putative NLPs were identified for further functional evaluation. On the basis of sequence homology analysis, we found that each of three genes (*PcNLP*13, *PcNLP*14, *PcNLP*15) is also a fragment of a single longer *NLP* gene, and *PcNLP*5 is a fragment of *PcNLP*2. Here, we cloned the full-length of *PcNLP*13, *PcNLP*14, and *PcNLP*15, whereas previously we cloned only fragments of them [51]. Amino acid sequences were deduced from the open reading frames; none of them has an intron. The protein sequences of these PcNLPs were submitted to SignalPv4.0 (http://www.cbs.dtu.dk/services/SignalP/) for secreted signal peptide prediction. PcNLP1*,* PcNLP2*,* PcNLP3*,* PcNLP6-10, PcNLP16, and PcINF1 had a signal peptide consisting of 17 to 22 amino acid residues (Table 1), which was predicted to regulate the secreted proteins. The other PcNLP did not have a predicted signal peptide and therefore may not secrete the PcNLPs proteins into the apoplast in native mycelia. Nine were single copy while the rest have from 2–6 copies each, and *PcINF*1 has 19 copies (Table 1). Notably, four other *PcNLP* genes (*PcNLP*4, *PcNLP*11, *PcNLP*12, and *PcNLP*16) have the restriction enzyme sites as predicted with DNAMAN, thus, these four genes were not compatible for functional analysis. Moreover, the mRNA expression of 11 genes can be detected by RT-PCR (data not shown), indicating that their transcripts are present. Thus, only 11 genes were selected for functional analysis during infection by *P. capsici* (Table 1).

**Table 1:** **The data of 15 *PcNLP *** **genes and *PcINF*** **1 from *P. capsici *** **SD33**

**Genes**	**GenBank No**	**Extracellular protein/Signal peptide length**	**SignalP length**	**Protein molecular weight (kDa)**	**Multicopy of each gene in JGI of *P. capsici *** **genome**
*PcNLP*1^*^	HM543167	Y	18	25.6	70849, 23286, 7756, 82067
*PcNLP*2^*^	HM543168	Y	19	26.9	23292, 7613, 37194,70852, 23292, 7613, 122619, 37194
*PcNLP*3^*^	HM543169	Y	19	25.4	71103, 23660, 7723, 82430, 116399
*PcNLP*4	HM543170	N	0	15.3	65858, 41937, 41936, 41935, 41934
*PcNLP*6^*^	HM543172	Y	19	37.3	24573
*PcNLP*7^*^	HM543173	Y	19	35.0	68295
*PcNLP*8^*^	HM543174	Y	18	34.4	26658, 8415
*PcNLP*9^*^	HM543175	Y	17	35.1	68297
*PcNLP*10^*^	HM543176	Y	17	25.5	70850, 23459, 1237
*PcNLP*11	HM543177	N	0	30.8	20844
*PcNLP*12	HM543178	N	0	29.8	21024
*PcNLP*13^*^	HM543179	N	0	29.7	123779
*PcNLP*14^*^	HM543180	N	0	27.5	9358
*PcNLP*15^*^	HM543181	N	0	30.7	108409
*PcNLP*16	HM543182	Y	19	37.1	107869
*PcINF*1	JX948084	Y	22	12.20	70621, 81778, 55432, 55431, 55430, 55429, 55428, 55427, 55426, 55425, 55424, 55423, 55422, 23123, 22825, 9413, 9410, 122465, 116044

A signal peptide of the PcNLPs is predicted with the tool SignalP4.0. The SignalP Network predicted cleavage site between 17 and 22 amino acid residues.

^*^The *PcNLP* genes were selected for functional analysis. ‘Y’ has a signal peptide. ‘N’ has no a signal peptide.

On the basis of sequence homology analysis, these 11 PcNLPs shared a conserved GHRHDWE motif and a relatively conserved hexapeptide QDLIMW at the C-terminal end. These characteristics identify any new peptide sequence as an NLP. Each *PcNLP* gene has four potentially coding residues that most likely correspond to the residues existing in the crystal structure of an NLP of *Pythium aphanidermatum*[39]. These residues were numbered as D^112^, H^120^, D^123^, and E^125^ in the PcNLP1 structure (Additional file 1: Figure S1).

**Figure 1:**
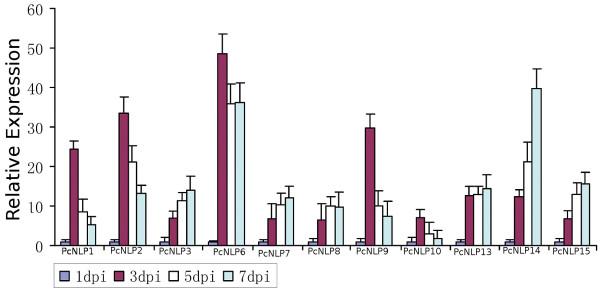
**RT-PCR analysis of 11 *PcNLP *genes expression patterns in inoculated pepper leaves*.*** Accession numbers are *PcNLP*1, *PcNLP*2, *PcNLP*3, *PcNLP*6, *PcNLP*7, *PcNLP*8*, PcNLP*9, *PcNLP*10, *PcNLP*13, *PcNLP*14, and *PcNLP*15. The *β-Actin*, *β-Tublin* and *Ubc* of *P. capsici* and *β-Actin* of pepper were used for endogenous controls. Data represent the average of three independent experiments with standard errors.

**Figure 2:**
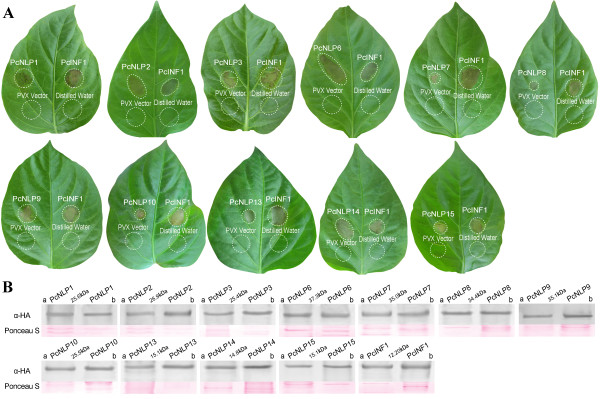
**Comparative analysis of necrosis-inducing response observed for various *PcNLP *genes by PVX agroinfection in pepper leaves. A**: Leaves were inoculated with *A. tumefaciens* harboring the PVX vector containing the respective *PcNLP* genes. Accession numbers are *PcNLP*1, *PcNLP*2, *PcNLP*3, *PcNLP*6, *PcNLP*7, *PcNLP*8, *PcNLP*9, *PcNLP*10, *PcNLP*13, *PcNLP*14, and *PcNLP*15. Each leaf was co-inoculated with *PcINF*1 at symmetric sites as positive control. The empty PVX vector and distilled water used as negative controls. Circles indicate the disservice regions around the point of inoculation. Photographs show individual, representative leaves taken at 10 dpi. The experiments were repeated at least three times. **B**:Western blot analysis of each *PcNLP* gene secreted following PVX vector agroinfection in pepper leaves. The proteins of agroinfiltrated leaves expressing *PcINF*1 with HA-tag and each *PcNLP* gene secreted from strain SD33 were used as positive controls. The total proteins of wild type leaves were used as negative control. **a**: The protein extracted from lesions spots; **b**: The protein extracted from strain SD33.

The mRNA expression of many *PcNLP* genes during infection by *P. capsici* has not been examined previously. In order to determine the expression patterns of these 11 targeted *PcNLP* genes at different time points after infection using zoospores, we performed qRT-PCR analysis. We used a cycle threshold (CT) cut-off value (>28) as a detection limit, and none of these 11 targeted genes produced CT values below this threshold at any of the sampling points. The qRT-PCR melting curve of each *PcNLP* gene was amplified by specificity of the qRT-PCR primers as shown in Additional file 2: Figure S3. Pepper leaves showed different degrees of lesion formation at different post-inoculation times (Data not shown). Water-soaked lesions were first observed at 1 to 2 days post-inoculation (dpi). The leaf lesions gradually expanded around the inoculation point from 1 to 7 dpi. Within 3 dpi necrotic areas in the lesions were noted, and the lesions were nearly completely rotten at 5 dpi. After 7 dpi, the mRNA could still be extracted from parts of the lesions but after 10 dpi the mRNA could not be extracted from the lesions. Total mRNA was only extracted from frozen lesions at 1, 3, 5, and 7 dpi, or from filtered mycelium of wild-type strain SD33 using the TRIZOL procedure. Thus, it was impossible to analyze mRNA expression levels related to infection time up to 10 dpi by qRT-PCR. Three housekeeping genes of *P. capsici* and pepper were used as constitutive expression internal controls and were used jointly as a reference to the microarray data of qRT-PCR detection. Figure 1 shows mRNA expression patterns of 11 *PcNLP* genes between the two experimental assays using qRT-PCR. Five (*PcNLP*1*, PcNLP*2, *PcNLP*6, *PcNLP*9, *PcNLP*10) reached the highest expression levels at 3 dpi, followed by a gradual decline, especially noted for *PcNLP*6, which showed the greatest expression over the period of 3 to 7 dpi. The expression of five other genes (*PcNLP*3, *PcNLP*7, *PcNLP*13, *PcNLP*14, *PcNLP*15) gradually increased to a maximum at 7 dpi; of these, *PcNLP*14 was expressed at the highest levels. Therefore, *PcNLP*2, *PcNLP*6, and *PcNLP*14 were, overall, the most strongly expressed during infection by *P. capsici*. In Figure 2A, from 3 to 7 dpi, these 11 *PcNLP* genes are classified into different transcription types based on their average induction levels. Also, *PcNLP*2, *PcNLP*6, and *PcNLP*14 showed the highest expression levels but *PcNLP*1 and *PcNLP*9 showed higher transcription levels than the six other genes. In contrast, six other genes (*PcNLP*3, *PcNLP*7, *PcNLP*8, *PcNLP*10, *PcNLP*13, *PcNLP*15) showed low transcription levels, especially, three (*PcNLP*7, *PcNLP*8, *PcNLP*10) showed the lowest transcription levels. All of these data indicate that these 11 targeted *PcNLP* genes are expressed at different levels at different times and contributed to different transcription types on the mRNA expression levels, suggesting that each targeted gene affects cells or tissues differently depending on the stage of infection.

### Functional analysis of *PcNLP* genes by PVX vector agroinfection assay in pepper and tobacco plants

**Figure 3:**
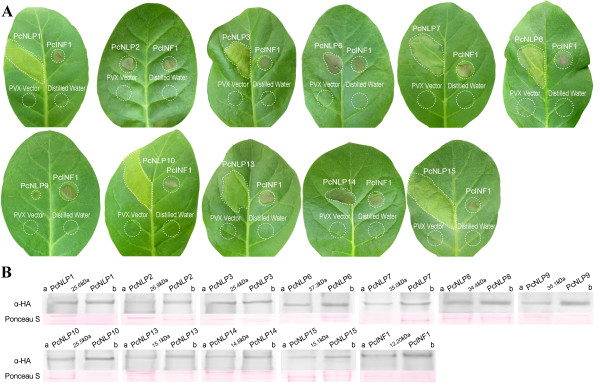
**Comparative analysis of chlorosis-inducing or necrosis response observed for various *PcNLP *genes by PVX agroinfection in tobacco leaves. A**: Leaves were inoculated with *A. tumefaciens* harboring the PVX vector containing various *PcNLP* genes. Accession numbers are *PcNLP*1, *PcNLP*2, *PcNLP*3, *PcNLP*6, *PcNLP*7, *PcNLP*8, *PcNLP*9, *PcNLP*10, *PcNLP*13, *PcNLP*14, and *PcNLP*15. Each leaf was co-inoculated with *PcINF1* at symmetric sites as positive control. The empty PVX vector and distilled water were used as negative controls on the same leaf. Circles indicate the disservice regions around the point of inoculation. Photographs show individual, representative leaves taken at 10 dpi. The experiments were repeated at least three times. **B**: Western blot analysis of each *PcNLP* gene secreted following PVX vector agroinfection in tobacco leaves. The proteins of agroinfiltrated leaves expressing *PcINF*1 with HA-tag and each *PcNLP* gene secreted from strain SD33 were used as positive controls. The total proteins of wild type leaves were used as negative control. **a**: The protein extracted from lesions spots; **b**: The protein extracted from strain SD33.

To determine whether any of the targeted *PcNLP* genes are capable of inducing necrosis in *C. annuum*, the usual host of *P. capsici*, and *N. benthamiana* which is not normally a host of this pathogen, we agroinfiltrated host cells with a PVX vector pGR106 [57] that carried each of the *PcNLP* genes from which a predicted signal peptide was not removed. In fact, some *PcNLP* genes contained sequences of the native signal peptide which secreted PcNLPs proteins from cytoplasm into apoplast of the mycelium. In this case, however, all these targeted *PcNLP* genes produced directly the secretion PcNLPs proteins in plant cytoplasm or apoplast after being agroinfiltrated into the plant tissue with PVX vector, where they functioned to degrade plant cell walls (Figures 2A and 3A). This is not related to the presence or absence of a signal peptide in the PcNLP when they are agroinfiltrated into the plant tissue. Each leaf was simultaneously inoculated with *PcINF*1, empty-vector, and distilled water. Representative phenotypes of all tested *PcNLP* genes and *PcINF*1 are shown in Figures 2A and 3A.

The diameter of necrotic spots in both plants was significantly larger when *PcINF*1 was injected than those of each targeted *PcNLP* gene. The results were consistent with previous results [18,53]. Notably, the degree of symptom development in pepper leaves in response to each *PcNLP* gene was noticeable elevated compared with the response in tobacco leaves. At the same time, the necrotic response in pepper leaves caused by *PcINF*1 was stronger than that in the tobacco leaves (Figures 2A and 3A). The empty-vector pGR106 and distilled water control did not induce any chlorosis or necrosis in either plant. This experiment demonstrated that the induction of most targeted *PcNLP* genes could trigger chlorosis or necrosis in leaves of pepper or tobacco independently of the *PcINF*1 gene.

In our experiments, each targeted PcNLP with an HA tag was associated with a distinct chlorotic or necrotic response in *C. annuum* and *N. benthamiana* (Figures 2A and 3A). In order to further determine the necrosis-inducing activity of the *PcNLP* genes, Western blot was used to determine whether the ability to induce chlorosis or necrosis was associated with the expression of the PcNLP proteins. The total proteins of agroinfiltrated leaves expressing PcNLP or PcINF1 with an HA-tag were extracted for western blot experiments. Western blots revealed that all of the PcNLP proteins and PcINF1 are detectable in the lesions of *C. annuum* and *N. benthamiana* (Figures 2B and 3B), but none of the *PcNLP* genes were detectable in the wild-type leaves (data not shown). Surprisingly, only three (*PcNLP*2, *PcNLP*6, *PcNL*P14) caused the largest necrotic areas in both hosts (*C. annuum* and *N. benthamiana*) at 7 dpi (Figures 2A and 3A), suggesting that these three genes could contribute strongly to virulence during infection by *P. capsici*. In the leaves of *C. annuum*, the expression of three genes (*PcNLP*1, *PcNLP*3, *PcNLP*9) induced distinct chlorosis at 3 dpi (data not shown), and all the chlorotic areas gradually turned brown and became moderately necrotic at 7 dpi (Figure 2A). The expression of two other genes (*PcNLP*13, *PcNLP*15) caused only small yellow areas at 3 dpi; these areas expanded somewhat and became necrotic at 7 dpi (Figure 2A). There was no visible reaction of *C. annuum* to *PcNLP*7, *PcNLP*8, and *PcNLP*10 for several days, but by 7 dpi small necrotic lesions were visible (Figure 2A). In *N. benthamiana*, the expression of *PcNLP*2, *PcNLP*6, and *PcNLP*14 caused strong necrosis at 7 dpi, similar to what was seen in *C. annuum* at 7 dpi (Figure 3A), and the expression of *PcNLP*9 caused only small necrotic areas at 7 dpi (Figure 3A). Seven genes (*PcNLP*1, *PcNLP*3, *PcNLP*7, *PcNLP*8, *PcNLP*10, *PcNLP*13, *PcNLP*15) only resulted in chlorotic areas, without necrosis at 7 dpi (Figure 3A). The smallest chlorotic areas were induced by *PcNLP*3 at 7 dpi, and the chlorotic areas caused by *PcNLP*1, *PcNLP*7, *PcNLP*10, and *PcNLP*15 were larger than those caused by *PcNLP*8 and *PcNLP*13 (Figure 3A). Therefore, the members of the *PcNLP* family are similar to *PcINF*1 in their ability to induce chlorosis or necrosis during infection of pepper and tobacco, but the necrotic or chlorotic response caused by the targeted *PcNLP* genes and *PcINF1* was stronger in pepper leaves (the usual host) than in tobacco leaves (an unusual host) (Figures 2A and 3A). In Figures 2B and 3B, all 11 *PcNLP* genes showed different toxicity on leaves of *C. annuum* and *N. benthamiana* within 7 days of agroinfiltration. In summary, *PcNLP*2, *PcNLP*6, and *PcNLP*14 always induced the strongest toxicity on the leaves of both hosts by 7 dpi, but eight other genes induced low toxicity on the leaves of both hosts by 7 dpi (Figures 2A and 3A). However, *PcNLP*1 and *PcNLP*9 induced higher toxicity on leaves of *C. annuum* than that of the six other genes (*PcNLP*3, *PcNLP*7, *PcNLP*8, *PcNLP*10, *PcNLP*13, *PcNLP*15) by 7 dpi (Figure 2B). In contrast, these six other genes induced low toxicity on leaves of *C. annuum*, especially, *PcNLP*7, *PcNLP*8, and *PcNLP*10 which induced the lowest toxicity by 7 dpi.

These results demonstrated that most of the members of the *PcNLP* family can express in host *C. annuum* and non-host *N. benthamiana* plants by triggering chlorotic or necrotic responses. They further suggest that individual *PcNLP* genes have different phytotoxic effects during infection by *P. capsici*, but that *PcNLP*2, *PcNLP*6 and *PcNLP*14 may play important roles in symptom development and may be crucial for virulence and necrosis-inducing activity or cell death. Moreover, the PcNLPs can trigger a disease response in tobacco but the effect in this non-host was muted when compared to the response in the usual host.

### Site-directed mutation of PcNLP1

**Figure 4:**
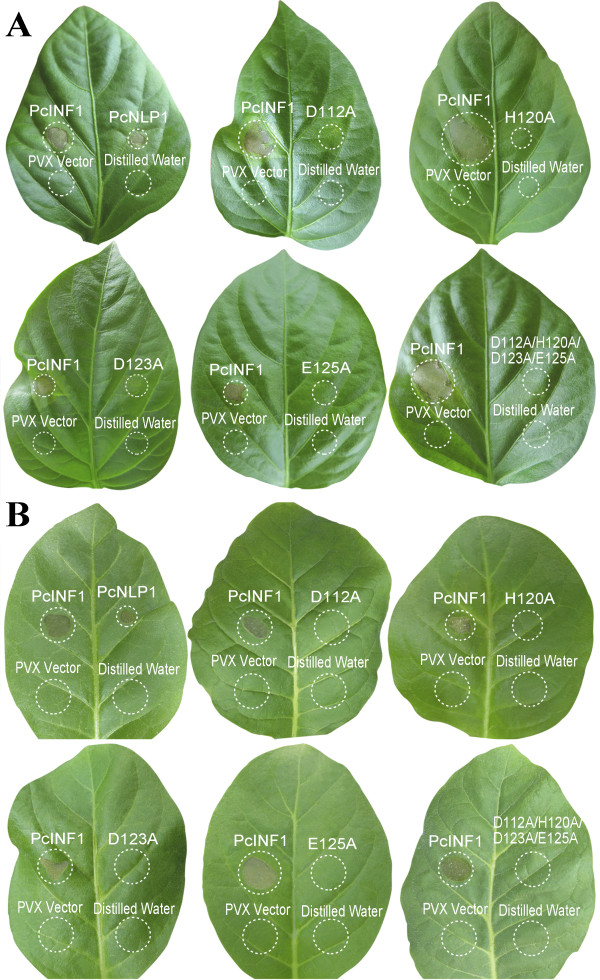
**Response of pepper leaves (A) and tobacco leaves (B) individually inoculated with *A. tumefaciens *harboring PVX vector containing various site-directed mutations of *PcNLP*1.** Accession numbers are *PcNLP*1, *PcNLP*1D112A, *PcNLP*1H120A, *PcNLP*1D123A, *PcNLP*1E125A, and *PcNLP*1D112A/H120A/D123A/E125A. Each leaf was co-inoculated with *PcINF*1 at symmetric sites as positive control. The empty PVX vector and distilled water were used as negative controls. Circles indicate the disservice regions around the point of inoculation. Photographs show individual, representative leaves taken at 10 dpi. The experiments were repeated at least three times.

PcNLP1 was chosen for site-directed mutagenesis because its expression levels were similar to those of other PcNLPs. It provided a readily identifiable phenotype in pepper and tobacco leaves, and it was one of the proteins that were predicted to be secreted during infection. To further confirm the functions of PcNLP *in vitro*, we created five mutations in PcNLP1 (D112A, H120A, D123A, E125A, D112/H120/D123/E125A) and constructed a PVX vector for each mutated residue. The ability to induce necrosis or cell death was tested on tobacco and pepper plants by agroinfection with PVX in the same manner as previously described. None of the mutated residues triggered a hypersensitive response in leaves of either plants after 7 dpi. Representative phenotypes of the five mutations were shown in Figure 4A, B. PcINF1 and unmutated PcNLP1 always induced a necrotic response around the point of inoculation of leaves of both plants (Figure 4A, B). The empty-vector pGR106 and distilled water did not cause any response in leaves of either plant. These results indicated that these four conserved residues in the PcNLP1 protein (D^112^, H^120^, D^123^, and E^125^) were likely responsible for the induction of necrosis or chlorosis, and indicate that each of the amino acid mutations possesses the effect on regulating the active sites of the PcNLP1 protein, as well as those of other PcNLPs proteins. DNA sequences of *PcNLP*1, *PcNLP*1D112A, *PcNLP*1H120A, *PcNLP*1D123A, *PcNLP*1E125A, and *PcNLP*1D112/H120/D123/E125A were presented in supplementary materials (Additional file 3: Figure S2).

### Generation of stable transformation lines, qRT-PCR analysis and impaired virulence

**Figure 5:**
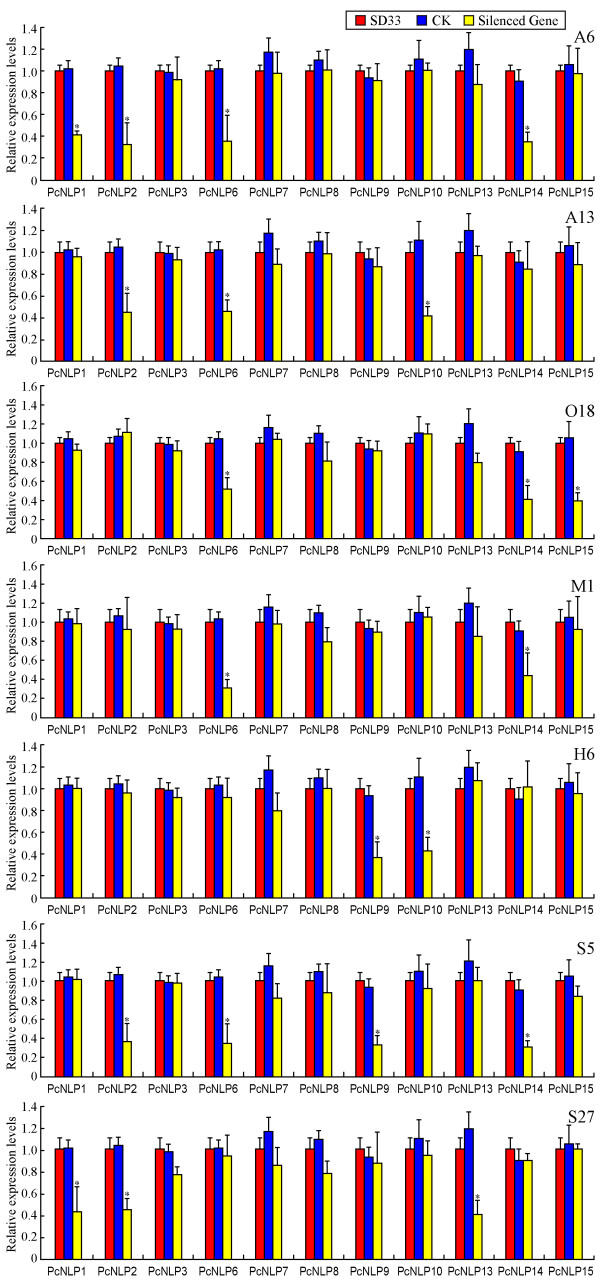
**qRT-PCR analysis expression levels of 11 PcNLPs in a set of silenced lines.** Each of the seven transformants (A6, A13, O18, M1, H6, S5 and S27) is presumed to contain different silenced genes. The asterisk is shown that the different silenced genes occurred simultaneously in each of the transformant line. From A6 to S27 showed the relative expression levels of a set of *PcNLP* genes in different transformant lines. Error bars represent confidence intervals calculated using three replicates for each sample. To allow comparisons of expression levels between genes, expression is shown as a value relative to the mean expression for all control lines. Three housekeeping genes *β-Actin*, *β-Tublin* and *Ubc*was were used for endogenous controls. WT (wild-strain SD33) and CK (a strain expressing only the selected gene) were used as positive controls. The experiments were repeated three times for the 11 *PcNLP* genes.

We attempted to develop stable transformations for each *PcNLP* gene through polyethylene glycol (PEG)-mediated protoplast gene-silencing [58]. A total of 86 putative transformant strains were grown on a selection medium with 50 μg/μl G418 (Sigma). Seven putative *PcNLP* transformant lines (A6, A13, O18, M1, H6, S5, S27) were obtained using RT-PCR detection (data not shown). The bands from the transformants were faint or missing when compared to *P. capsici* strains SD33 and CK. Each transformant line was initially expected to contain a trigger gene as follows: A6 (*PcNLP*2), A13 (*PcNLP*10), O18 (*PcNLP*15), M1 (*PcNLP*6), H6 (*PcNLP*9), S5 (*PcNLP*14), S27 (*PcNLP*13). In these experiments, we used the total length of each gene to be silenced. The observed patterns of silenced genes were unexpected. Several members in the *PcNLP* family were almost simultaneously silenced in each transformant line. As shown in Figure 5, the asterisk (*) indicates that the different silenced genes occurred simultaneously in each of the transformant lines. As is also shown in Figure 5, each transformant line contained several silenced genes, and each silenced gene was assigned to the transformant line. These results are possible only if all targeted genes shared a relatively high sequence similarity. In these seven transformant lines, no differences in growth rate, hyphal development, sporangial morphology or size, or numbers of zoospores released were observed when compared with SD33 and CK (data not shown).

**Figure 6:**
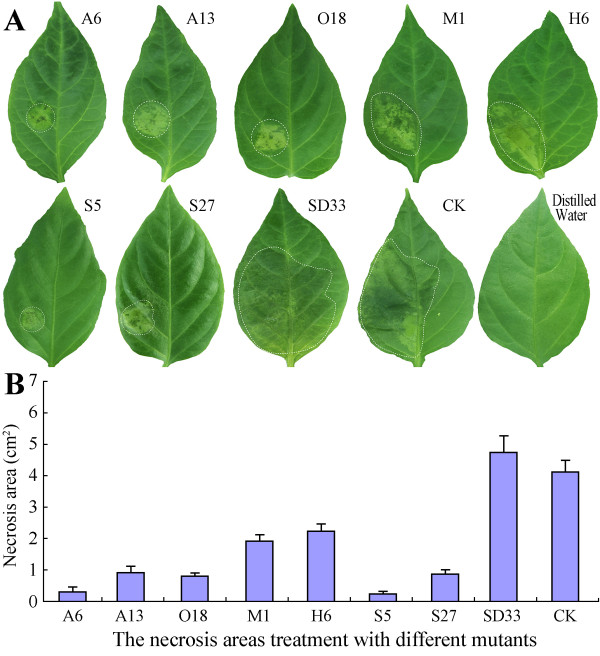
**Stable transformation lines with impaired virulence on pepper leaves. A**: Symptoms appearing on pepper leaves after inoculation with different transformants (A6, A13, O18, M1, H6, S5, S27); WT (strain SD33) and CK (a strain only expressing the selected gene) were used as positive controls; Distilled water was used as negative control. Circles indicate the disservice regions around the point of inoculation. The experiments were repeated three times for all strains. Photographs show individual, representative lesion areas taken at 3 dpi. **B**: Mean lesion areas appearing on pepper leaves inoculated with different strains. Bars represent the mean ± standard error of 14 leaves. The mean lesion areas were evaluated at 5 dpi.

**Figure 7:**
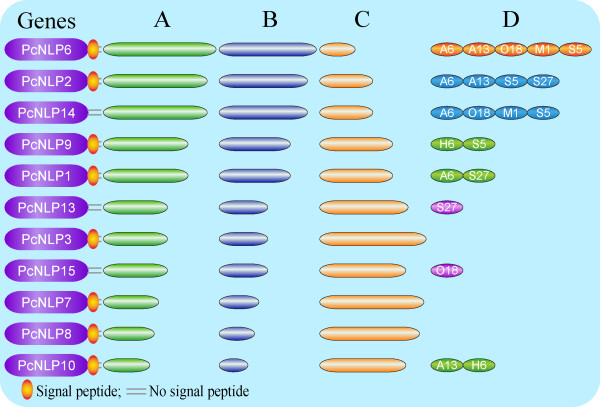
**A summary shows the relationships of the 11 *PcNLP *genes along with the transcriptional levels, the toxicity degrees, the silencing levels and the secretion signal status among 11 *PcNLP *genes. A**: The transcription levels of 11 *PcNLP* genes from 3 to 7 dpi during the pepper leaves infection by *P. capsici*. The more long of the green cylinder is represented that the transcriptional levels is more strongly from 3 to 7 dpi. **B**: Toxicity types of 12 *PcNLP* genes on leaves of *C. annuum* after agroinfiltration with PVX by 7 dpi. The more long of orchid cylinder is represented that the toxicity degree is more strong at 7 dpi. **C**: Silencing patterns of all targeted *PcNLP* genes in different silencing lines. The more short of the orange cylinder is represented that the silencing degree is more strongly among all targeted genes. **D**: Each transformant line contained several silenced genes, and assigned each silenced gene to several transformant lines.

To more directly evaluate the contribution of each *PcNLP* gene to *P. capsici* toxicity, we employed qRT-PCR to evaluate transient expression of each silenced gene in putative transformant lines compared with SD33 and CK, using RNAs extracted from growing mycelia. Three housekeeping genes, identified from the microarray data as constitutively expressed, were used jointly as a reference to relate to the microarray data of the qRT-PCR detection. The expression levels of individual genes varied in different silenced lines because silencing a targeted gene possibly caused repression of other members in the PcNLP family. Leaves of susceptible pepper (*C. annuum* inbred line 06221) were inoculated with zoospores to test the virulence of each transformed line. The infection efficiencies and the average areas of lesions were shown in Figure 6A and B. All pepper leaves treated with CK and SD33 showed large areas (4.25 to 4.95 cm^2^) of water-soaked lesions from 1 to 2 dpi; these are typical symptoms of *Phytophthora* foliar blight (Figure 6A: SD33 and CK). In contrast, inoculation with any of the seven silenced lines resulted in significantly smaller lesions (ca. 0.5-2.2 cm^2^) than those of SD33 and CK (*P* < 0.01). Inoculation with silenced lines A6 and S5 resulted in the smallest lesions (ca. 0.5 to 0.6 cm^2^, respectively) (Figure 6A, B). This result is directly correlated to strongly repressed expression of four *PcNLP* genes in these two silenced lines (Figure 5). In A6, expression of four genes (*PcNLP*1, *PcNLP*2, *PcNLP*6, *PcNLP*14) was very clearly reduced (60% to 68%), and in S5 expression of four genes (*PcNLP*2, *PcNLP*6, *PcNLP*9, *PcNLP*14) was similarly reduced (60% to 70%). Notably, *PcNLP*2, *PcNLP*6, and *PcNLP*14 were simultaneously repressed to a significant degree in A6 and S5. In consequence, the simultaneous repression of these three genes in *P. capsici* resulted in significantly reduced virulence for the pathogen (Figure 6 A6, S5). Otherwise, when treated with three other silenced lines (A13, O18 and S27), small lesions (ca. 0.9-1.0 cm^2^) developed on pepper leaves that were larger than those developing after inoculation with silenced lines A6 and S5 (*P* > 0.05). In each of these three lines (A13, O18, S27), three *PcNLP* genes were simultaneously silenced and showed a conspicuous reduction in expression of 50% to 60%. In A13, suppression of *PcNLP*2, *PcNLP*6, and *PcNLP*10 resulted in a reduction of expression by 54% to 60%. In O18, silencing of *PcNLP*6, *PcNLP*14, and *PcNLP*15 reduced expression by 50% to 58%. In S27, suppression of *PcNLP*1, *PcNLP*2, and *PcNLP*13 reduced expression by 50% to 60% (Figure 5). Thus, the *PcNLP* genes in A13, O18, and S27 are not repressed as strongly as the *PcNLP* genes in A6 and S5, which is possible to elucidate why these three lines resulted in slightly increased virulence compared with A6 and S5 (Figure 6A, B). These results indicate that the simultaneous silencing of a few *PcNLP* genes in a strain of *P. capsici* can result in significantly reduced virulence (Figure 6A, B). However, when treated with M1 and H6, large necrotic areas (ca. 1.8 and 2.2 cm^2^, respectively) developed that were two to six times larger than those seen on pepper leaves when treated with five other silenced lines (*P* < 0.05), but were at least two times smaller than those of SD33 and CK (Figure 6A, B). In these two silenced lines there was modest reduction (54%-65%) in expression of two *PcNLP* genes. In M1, the expression of *PcNLP*6 and *PcNLP*14 was reduced by 55% to 65%, and in H6 expression of *PcNLP*9 and *PcNLP*10 was reduced by 57% to 63%. In these two lineages, nine other genes retained high expression levels, near those of SD33 and CK (Figure 6). As the result, when only two *PcNLP* genes in M1 or H6 were slightly repressed, virulence of these two lines was slightly increased in comparison with five other lines (Figure 6A, B). However, the expression of *PcNLP*3 was only reduced by 15% in line S27, the reduced expression of *PcNLP*7 ranged 10% to 12% in two lines (A13 and S27), and expression of *PcNLP*8 was only slightly reduced in three lines (S27, M1, and O18). In Figure 7C, the various degrees of silencing of each targeted gene in different lines are illustrated, and the shortest of the orange cylinders indicates a greater degree of silencing for each of the targeted genes.

Taken together, the above data reveal that the degree of virulence of different silenced lines is correlated with the repression of the *PcNLP* genes and the consequent suppression of their expression levels. The repression and expression of the targeted *PcNLP* genes in silenced lines was variable and showed that ectopic expression of some targeted genes with the heterologous promoter caused mRNA expression levels to be several-fold lower in silenced lines than those in the controls. These results suggested that the variability in expression of *PcNLP* genes in the different silenced lines probably results from an eligible or ineligible position effect of the introduced DNA within the *P. capsici* genome. In the present study, the expression of *PcNLP*2, *PcNLP*6, and *PcNLP*14 was strongly repressed in more silenced lines than those of any other genes. *PcNLP*6 was significant silenced in five lines A6, A13, O18, M1, S5, similar to the expression of *PcNLP*2 and *PcNLP*14, which was strongly silenced in four lines. Therefore, these three genes were effectively silenced compared to other members in the PcNLP family. In the lines A6 and S5, moreover, *PcNLP*2, *PcNLP*6, and *PcNLP*14 were highly repressed, which was parallel to the significant reduction in necrotic response after infection of leaves of pepper and tobacco. In the lines O18 and M1, however, the suppressed genes included *PcNLP*6 and *PcNLP*14, but the expression of *PcNLP*2 is similar to SD33 and CK. As a result, both O18 and M1 showed slightly increased virulence when compared to A6 and S5. Therefore, the simultaneous presence of *PcNLP*2, *PcNLP*6, and *PcNLP*14 may be required for a complete necrotic response during *P. capsici* infection, suggesting that these three *PcNLP* genes might be more closely linked to the necrotic response than other members in the *PcNLP* family and might be crucial for virulence and necrosis-inducing activity during *P. capsici* infection.

## Discussion

Since an NLP was identified in the vascular wilt fungus *Fusarium oxysporum*[22], NLPs have been predicted to occur in a great variety of microbes including bacteria, fungi and stramenopiles [28,59]. NLPs are common and numerous in several stramenopile genomes [28]. We identified 18 NLP paralogs (*PcNLP*1 to *PcNL*P18) from *P. capsici* SD33 [51]. The conserved motif GHRHDWE is always located in the central region of those PcNLPs, and two cysteine residues in the N-terminal position of the PcNLP are essential for biological activity. In these respects, PcNLPs are similar to those in *P. megakarya*, *P. parasitica*, *P. sojae*, and *Hyaloperonospora arabidopsidis*[17-21]. Thus, the NLPs family of effectors appear to be highly conserved across the genus *Phytophthora* indicates that it may play an important and conserved role in all species.

To analyze the function of the PcNLP members as toxins responsible for symptom development and cell death, we evaluated the function of 11 *PcNLP* genes on active transcripts *in vitro* and *in vivo* in leaves of pepper and tobacco. We further detected the function of PcNLPs protein *in vitro* based on the site directed mutagenesis of four amino acid residues in a conserved motif. The qRT-PCR analysis allowed for the detection and quantification of the transcriptional changes of the 11 *PcNLP* genes in a series of *P. capsici*-infected pepper leaves at distinct phases of the plant/pathogen interaction. Five (*PcNLP*1, *PcNLP*2, *PcNLP*6, *PcNLP*9, *PcNLP*10) achieved peak expression early, at three days following infection. This pattern is similar to the reported expression profiles reported of NLPs in *Moniliophthora perniciosa* and *Phytophthora sojae*, where peak expression was associated with the appearance of disease symptoms in the initial stage of the interaction [18,32]. Six (*PcNLP*3, *PcNLP*7, *PcNLP*8, *PcNLP*13, *PcNLP*14, *PcNLP*15) gradually increased their expression levels, peaking at a late phase of the infection. The pattern of expression has not been observed previously. As shown in Figure 1, the various expression patterns of different *PcNLP* genes in pepper tissues enable us to speculate about their contributions to differences in pathogenicity or virulence during *P. capsici* infection. Symptomatic response to different *PcNLP* genes was related to variation transcription levels *in vivo* during infection by *P. capsici*. Four (*PcNLP*2, *PcNLP*6, *PcNLP*9, and *PcNLP*14) induced the most severe symptom development in pepper or tobacco leaves (Figures 2A and 3A) and showed high transcription levels during infection (Figure 1). In contrast, six (*PcNLP*3, *PcNLP*7, *PcNLP*8, *PcNLP*10, *PcNLP*13, *PcNLP*15) were transcribed at low levels (Figure 1), which were linked to weak symptom development in both tested plants. These combined patterns have been observed previously for other hosts and their parasites. For example, the peak expression of *P. sojae* NLPs was directly related to the occurrence of disease symptoms in infected plants as the pathogen transitioned from the biotrophic to the necrotrophic growth state [18], while *MgNLP* of the fungal pathogen *Mycosphaerella graminicola* appeared to be highly expressed specifically at the end of the symptomless phase of infection of wheat leaves [32]. The strong expression in plants of some PcNLPs and their multi-copy status in the genome enabled us to answer a difficult question for this pathosystem where *NLP* genes exist in multiple copies, namely; are *NLP* genes major virulence factors for the pathogenic lifestyle of *P. capsici*?. In our analysis, *PcNLP*2, *PcNLP*6, and *PcNLP*14 were proposed to play a crucial role in promoting virulence and inducing necrosis or cell death. Other organisms have provided strong evidence for their function as virulence factors with the characterization of NLPs in *Colletotrichum coccodes*[34] and *Erwinia carotovora* subsp. *carotovora*[12,13]. On the other hand, the NLP genes in pathogens such as *F. oxysporum* f. sp. *erythroxyli* and *Mycosphaerella graminicola* do not appear to affect their virulence [31,32], and the NLPs, *Bcnep*1 and *Bcnep*2 are apparently not related to virulence during *Botrytis cinerea* infection [33]. Thus, the members of the NLP families from different pathogens encode functionally different phytotoxic proteins that appear to perform a variety of functions during infection and produce variable extended phenotypes. The reasons for this phenomenon are unclear; however, recent data suggest that the effector proteins of many pathogens including *Phytophthora* species are under positive selection and are often considered to operate at the forefront of evolution in host-microbe interactions [37,60]. In addition, the failure to detect expression of 11 *PcNLP* genes at an early stage of infection is similar to the situation involving *PsojNIP* transcripts during the transition from biotrophy to necrotroph after infected by *P. sojae*[18]. This suggests that, as in *P. sojae*[20], some PcNLPs initiate the process of infection, but some other PcNLPs play important roles after the initiation of infection. These results might be due to differences in regulation, but it is likely that these genes have distinct functions during infection by *P. capsici* unrelated to the initiation of infection.

As described above, the expression patterns of all 11 *PcNLP* genes are shown in Figure 7A. Similar to other *Phytophthora* species, there are multiple copies of NLPs in the genome of *P. capsici* and the PcNLPs most likely perform different roles during the infection process. Overall, we conclude that *PcNLP* genes not only participate in inducing cell death and symptom development but also perform different roles at different phases of infection. In addition, these 11 *PcNLP* genes are linked to symptom development in pepper and tobacco, but the intensity of the symptoms was much more conspicuous in pepper, the usual host of *P. capsici*, than those in tobacco (Figures 2A and 3A). Similar variation in host-dependent symptom development in relation to NLPs from *P. sojae* and the fungus *Moniliophthora perniciosa* has been observed [21,26]. The availability of heterologously expressed PcNLPs allowed us to examine other characteristics of this protein. We were able to confirm that *PcNLP* genes encode chlorosis/necrosis-inducing proteins in leaves of pepper and tobacco, and that these proteins also stimulate the expression of the host’s defense-related genes in tissues of both plants. NLPs have been suggested to have dual functions in plant pathogen interactions: acting both as triggers of defense responses and as toxin-like virulence factors. Here, six *PcNLP* genes showed low transcription levels corresponding to weak symptom development, suggesting that these NLPs may stimulate immunity-associated defenses or act as triggers of immune responses in plants. These findings call for additional research.

We confirmed that four conserved amino acids (D112, H120, D123, and E125) in the putative active site and conserved motif have the ability to regulate the function of PcNLP1 (Figure 4A, B). This suggests that these four conserved amino acids provide similar function in paralogs. This is in agreement with previous studies in *P. aphanidermatum*[39].

In our study, it was difficult to identify isolates in which one targeted gene was silenced alone or all targeted genes were silenced simultaneously. This phenomenon was also observed in the silencing of six hydrophobins in *Cladosporium fulvum*[61]. Most members in the PcNLP family were not completely silenced but instead were suppressed different degrees. This may be related to the low expression levels of genes in the PcNLP family, or may be related to the difficulty of complete silencing in diploid stramenopiles. Three *PcNLP* genes (*PcNLP*2, *PcNLP*6, *PcNLP*14) (Figure 1) showing high expression during *P. capsici* infection were more down-regulated than other tested genes showing lower expression levels, strongly supporting that highly expressed genes are easier to suppress [62]. Several genes (*PcNLP*3, *PcNLP*7, *PcNLP*8, *PcNLP*9, *PcNLP*10, *PcNLP*13, *PcNLP*15) were linked to a weak necrotic response in plants, but their transformants showed various degrees of reduction. However, the expression levels of the three most similar paralogs (*PcNLP*1, *PcNLP*3, *PcNLP*10) were not significantly decreased in different transformants, but three other more divergent paralogs (*PcNLP*2, *PcNLP*6, *PcNLP*14) were always effectively silenced in several transformants. This corresponds to the results of Wroblewski et al*.*[63] in which the members of the NBS–LRR gene family showed similar patterns of silencing. In our experiments we targeted relatively large segments of the *PcNLP* genes (399–1017 bp). This indicates that the size of the silenced plasmid (pHAM34) is not limiting and that it will be feasible to assay multi-interfering constructs. It may also be feasible to interfere with several related genes with conserved domains; permitting coordinated suppression of a gene family [64]. In our study, necrotic lesions observed for several transformants were significantly smaller than those observed for the control strains. This suggests that individuals PcNLP may have an effect on its ability to establish infection on plant. Several studies have considered the function of NLP genes, and most conclude that several NLPs are indispensable for fungal infection [33,65]. Our study concluded that *PcNLP*2, *PcNLP*6, and *PcNLP*14 contribute greatly to the induction of necrosis during infection by *P. capsici*, and suggested that the simultaneous presence of *PcNLP*2, *PcNLP*6, and *PcNLP*14 may be required for a complete necrotic response.

Our results suggest that some PcNLPs play important roles in necrosis-inducing or pathogenicity during *P. capsici* infection. However, many aspects of *Phytophthora* pathogenicity remain obscure, and investigating the action of specific genes in the infection process has always been an arduous undertaking. However, elucidating the important role of pathogenicity genes in *P. capsici* will help advance understanding of the biology and pathogenicity of *Phytophthora* and other stramenopiles on diverse host plant species.

We found that each targeted *PcNLP* gene affects cells or tissues differently depending upon the stage of infection. Most *PcNLP* genes could trigger necrotic or chlorotic responses when expressed in the host *C. annuum* and the non-host *N. benthamiana*. Moreover, our results showed that individual *PcNLP* genes have different phytotoxic effects, but *PcNLP*2, *PcNLP*6, and *PcNLP*14 may play important roles in symptom development and may be crucial for virulence, necrosis-inducing activity, or cell death during infection by *P. capsici*.

## Conclusions

We found that each targeted *PcNLP* gene affects cells or tissues differently depending upon the stage of infection after inoculation with zoospore suspension of highly virulent *P. capsici* SD33 using qRT-PCR. Most *PcNLP* genes could trigger necrotic or chlorotic responses when expressed in the host *C. annuum* (inbred line 06221) and the non-host *N. benthamiana* after agroinfiltration the host cells of both plants with *A. tumefaciens* PVX vector carrying each of the *PcNLP* genes on evaluation of the necrotic response and the PcNLP proteins expression levels in the lesions of both plants. Otherwise, we obtained seven putative *PcNLP* silenced lines that was initially expected to contain a trigger gene, however, each of the silenced lines contained several silenced genes, and different silenced genes were assigned to the different silenced lines. On the evolution of the virulence of different silenced lines and the mRNA expression levels of different *PcNLP* genes, *PcNLP*2, *PcNLP*6 and *PcNLP*14 may be required for a complete necrotic response during *P. capsici* infection. Therefore, our results showed that individual *PcNLP* genes have different phytotoxic effects, but *PcNLP*2, *PcNLP*6, and *PcNLP*14 may play important roles in symptom development and may be crucial for virulence, necrosis-inducing activity, or cell death during infection by *P. capsici*.

## Methods

### Pathogen strain, plant cultivation and candidate gene selection

Highly virulent *Phytophthora capsici* strain SD33 has been tested in our laboratory and routinely cultured on 10% V8-juice agar medium at 25°C [51,66,67]. Production of sporangia and zoospores were performed as previously described [67].

A susceptible cultivar of pepper (*Capsicum annuum* inbred line 06221), and tobacco (*Nicotiana benthamiana*) were selected from different inbred lines based on evaluation of pathogenicity after inoculation with zoospores of highly virulent *P. capsici* SD33. This experiment was repeated over 3 years under controlled conditions and symptom development was documented. Seeds were germinated following surface-sterilization by immersion in sodium hypochlorite (0.5% vol/vol) for 30 min followed by thorough rinsing in sterile water. The seedlings were cultured in a tray containing heat-sterilized soil/sand (1:1) mixed at 25-28°C (16 h light period) in a growth chamber. The light intensity in the chamber was 300 and 450 mol m^-2^ s^-1^, which is the intensity that promotes greatest leaf expansion. Single seedlings at the three leaf stage were then transplanted into small plastic trays, and grown for 14 days under the same conditions [42].

The *PcNLP* genes were identified in the *P. capsici* reference genome by searching a six-frame translation of the genome in the DOE Joint Genome Institute database (JGI, http://img.jgi.doe.gov/cgi-bin/w/main.cgi) on the conserved GHRHDWE motif and then searching that subset for signal peptides with the tool SignalP4.0 (citation). Eighteen NLP-encoding genes (GenBank accession numbers HM543167 to HM543184) were identified from *P. capsici* SD33, RT-PCR detected expression for all but seven [51], of which 11 were selected for further functional analysis during *P. capsici* interactions with plants (Table 1).

To amplify the *PcINF*1 gene (GenBank accession number JX948084) from *P. capsici* SD33, pairs of primers (INF1F: 5′-ATGAACTTCCGTGCTCTGTTC-3′; INF1R: 5′-TTACAGCGACGCGCACGTGTT-3′) were designed using Primer Express 3.0 software based on sequences in the JGI database. Genomic DNA of SD33 was extracted from mycelium grown in 10% V8 liquid medium according to the protocol described by Tyler et al. [27]. Minor adjustments were made to PCR amplification as previously reported [51]. The PCR products were cloned in the T3-vector and confirmed by sequencing. Nucleotide and amino acid sequence homology searches were compared with the sequences in the NCBI-BLAST program according to previous reports [68]. The available PcNLPs amino acid sequences were aligned using Clustal X 2.0 [24]. Phylogenetic trees were generated by neighbor-joining, as implemented in PAUP*4.0 Beta (Sinauer Associates, Sunderland, MA, USA) with the default parameters. Nodal support of the trees was estimated by bootstrapping, with 1000 pseudoreplicate data sets.

### RNA extraction and SYBR green real-time RT-PCR assay

To monitor PcNLP transcript profiling during *P. capsici* infection of pepper, leaf inoculation using zoospores of *P. capsici* SD33 was performed as previously described [67]. Samples were collected at 1, 3, 5, and 7 days post infection (dpi) and put into liquid nitrogen immediately. Total RNA was extracted using the TRIZOL procedure (Invitrogen) from freeze-infected leaves, filtered mycelium grown in 10% V8-juice liquid medium at 25°C for three days, and from lesions infected by *P. capsici*. The RNA was quantified by measuring absorbance at 260/280 nm with a spectrophotometer and the quality was examined by electrophoresis on a 1.2% agarose gel containing formaldehyde. A total of 10 μg RNA was treated with 4 units of Rnase-free DNase (Takara) at 37°C for 30 min, and then used for reverse transcription with an Omniscript RT kit (Qiagen). The complete removal of all DNA was ratified using a PCR reaction run under the same conditions as those used for the RT-PCR, except for omission of the cDNA synthesis step.

For *PcNLP* transcript profiling analysis, SYBR green real-time PCR analyses were performed. Primers (Additional file 4: Table S1) were designed to anneal specifically to each targeted gene and three housekeeping genes *β-Actin*, *β-Tublin* and *Ubc* (ubiquitin C) of *P. capsici* and *β-Actin* of pepper [69] by using Primer 3.0 software for SYBR green real-time PCR (qRT-PCR). The *β-Actin*, *β-Tublin*, and *Ubc* genes were used as constitutively expressed endogenous controls and were used jointly as a reference to relate to the microarray data of the qRT-PCR detection. The expression of *PcNLP* genes in different lines was determined relative to the three reference genes followed by the ICycler IQ RT-PCR detection system (Bio-Rad, Denmark) and SYBR primer Script RT-PCR kit (TaKaRa, Japan). The 25 μl PCR reaction included 2.5 μl of cDNA template, 0.8 μM gene-specific primer for each *PcNLP* gene or housekeeping gene, 12.5 μL of 2 × SYBR Green PCR master mix, and 8.5 μL of distilled H_2_O. The reactions were performed on the ICycler IQ RT-PCR detection system (Bio-Rad, Denmark) under the following conditions: 95°C for 15 min; 40 cycles at 95°C for 10 s, 60°C for 15 s and 72°C for 30 s to calculate cycle threshold values; followed by a dissociation program of 79 cycles at 55°C to 95°C to obtain melt curves. The expression of each gene at 1 dpi was assigned the value of 1.0 to allow comparison between lines. The values of threshold cycles (CT) were ascertained automatically by instrument, and the fold changes of individual gene were calculated using the equation 2^- ΔΔCT^ according to revious descriptions [70]. The investigation was conducted twice, each with three independent biological replicates.

### Construction of recombinant *A. tumefaciens* binary PVX vectors

Candidate *PcNLP* genes were PCR amplified from genomic DNA of *P. capsici* SD33 using high-fidelity DNA polymerase (TakaRa Inc.) The primers (Additional file 5: Table S2) complementary to the 5′and 3′ends of each respective open reading frame were designed to include restriction site overhangs for cloning into PVX vector pGR106 [57]. Upstream primers contained sequences corresponding to the native signal peptide for extracellular targeting with the exception of *PcNLP*13, *PcNLP*14, and *PcNLP*15 for which their sequences do not encode the signal peptide. The PCR products were digested with appropriate restriction enzymes, size-fractionated and purified from 1.0% agarose gels prior to ligation into pGR106. Recombinant plasmids were maintained and propagated in *Escherichia coli* DH-5α with 50 μg/ml kanamycin and 12.5 mg/ml^-1^ tetracycline, grown in LB broth cultures for 48 h at 28°C. The cultures were centrifuged 10,000 g for 1 min. Each clone was verified by PCR using vector primers (forward: 5′-CAATCACAGTGTTGGCTTGC-3′, reverse: 5′-GACCCTATGGGCTGTGTTG-3′) and was then further checked by DNA sequencing. Plasmids were extracted from *E. coli* DH-5α and then were introduced into *Agrobacterium tumefaciens* GV3101 by electroporation. The transformants were selected on LB broth agar supplemented with 12.5 ppm tetracycline and 25 ppm kanamycin at 28°C. Plasmids obtained from the transformants and were tested by PCR for the presence of *PcNLP* gene insert. Individual colonies were toothpick-inoculated onto the lower leaves of *C. annuum* or *N. benthamiana* plants. Three days before infiltration, *A. tumefaciens* cells carrying *PcNLP* gene were inoculated into LB broth supplemented with tetracycline and kanamycin at 28°C for 48 h. The resultant cultures were prepared as method [57]. Infiltration involved use of a needleless 1-ml syringe placed against the lower side of the leaf. Each of the colony infiltration tests consisted of at least seven plants inoculated on three leaves. Colonies harboring *PcINF*1 [53] were infiltrated into symmetric sites on the same leaf and were used as positive control. The empty-vector and distilled water were used as negative controls. Routinely, infiltrations were performed on 5-week-old pepper leaves. Symptom development was monitored visually for 10 d after infiltration. Symptoms were scored and photographed at 7 d. All tests were carried out in three replicates.

### Protein extraction and western blot

The development of lesions in *C. annuum* and *N. benthamiana* was recorded visually 5 d after agro-infiltration by *Agrobacterium* cultures that carried the different *PcNLP* genes or *PcINF*1 with HA-tag, respectively. Western blots were done with tissue from 7 dpi lesions. The total proteins of lesion tissue of *C. annuum* or *N. benthamiana* were extracted by grinding 350 mg of 14 leaf lesions leaf or 14 wild leaves in 1 mL extraction buffer (50 mM Tris, pH 7.4, 150 mM NaCl and 1% Triton X-100) in the presence of 5 μL protease inhibitor cocktail (Sigma, P9599). Protein concentrations were determined by the Bradford method [71] using bovine serum albumin as a standard. Approximately 50 μg of total proteins was loaded on 12% SDS–PAGE gel using equivalent amounts of protein. After electrophoresis, proteins were transferred onto a polyvinylidene difluoride (PVDF) membrane (Millipore). Western blotting was carried out as previously described [72]. Mouse anti-HA monoclonal antibody (Sigma-Aldrich) and Goat anti-mouse IgG-peroxidase conjugate (Sigma-Aldrich) were used as the primary and secondary antibodies. The membrane was treated with Chemiluminescent Peroxidase Substrate-1 (Thermo Scientific Pierce, No. 34080, USA) for 2 min. The membrane was briefly drained and exposed to BioMax (Kodak, USA) light film several times (depending on results) for exposure signal development. The immunoblots were quantified using Quantity one software (Bio-Rad) and the chemoluminescence signal was imaged using a ChemiDoc XRS (Bio-Rad). Culture conditions for strain SD33 and the total proteins extractions were performed as reported previously [30]. The total proteins of lesions tissues of *C. annuum* and *N. benthamiana* agro-infiltrated expressing of *PcINF*1 with HA-tag and each *PcNLP* gene secreted from SD33 was used as a positive controls. Crude proteins from wild pepper or tobacco leaves were used as negative controls. Each experiment was repeated at least three times.

### Site-directed mutagenesis of *PcNLP1*

Based on the alignment of all *PcNLP* genes with reported *NLP* genes, *PcNLP*1, *PcNLP*2, *PcNLP*3, *PcNLP*6, *PcNLP*7, *PcNLP*8, *PcNLP*9, *PcNLP*10, *PcNLP*13, *PcNLP*14, and *PcNLP*15 showed high homogeneity to NLP_pya_ from *Pythium aphanidermatum,* and were presumed to have the putative active sites D112, H120, D123, and E125 (numbered according to each of these 11 *PcNLP* genes) [39]. These four conserved amino acids in *PcNLP*1 were individually exchanged for alanine using overlap PCR. The primers are listed in Additional file 6: Table S3. Also simultaneous substitution of all four amino acids by alanine was carried out to further investigate the characters of PcNLP proteins as described above [39]. All the mutants were verified by DNA sequence analysis. The mutants were analyzed for their ability to induce symptoms by agroinfiltration with PVX vector as described above. Each leaf was co-inoculated with *PcINF*1 at symmetric sites on the leaf. Both *PcNLP*1 and *PcINF*1 were used as positive controls. The empty vector pGR106 and distilled water were used as negative controls. The infiltrations were performed on 5-week-old pepper leaves or 4-week-old tobacco leaves. Symptom development was monitored visually 3 to 7 d after infiltration. Photographs were taken at 10 d. Each assay consisted of at least three plants inoculated on three leaves at least two different dates. The experiments were conducted with three replicates.

### Construction of recombinant plasmids for stable transformations of *P. capsici*

Strains of pHAM34 and pHspNpt were kindly provided by professor Wang Yuan Chao. Fragments for generating candidate constructs were amplified from cDNA and were digested with the restriction enzyme *Sma*I for cloning into the vector pHAM34. The resultant plasmids were verified by DNA sequence analysis. Primers used are in Additional file 7: Table S4. Both sense and antisense plasmids were used for transformation. Sub-cloning of *PcNLP* genes for orientation of *PcNLP* genes for transcription of the negative (anti-sense) strand was used for gene silencing. Stable transformation was fulfilled using the method of McLeod et al. [58] with the following modification: 2-d-old *P. capsici* mycelium, cultured in pea broth, was rinsed and washed in 0.8 M mannitol and then placed in enzyme solution (0.4 M mannitol, 20 mM KCl, 20 mM MES, pH 5.7, 10 mM CaCl_2_, 7.5 mg/mL lysing enzyme (Sigma-Aldrich L1412), and 3 mg/mL cellulase (Sigma-Aldrich C8546) and incubated for 40 min at 25°C with 10,000 g shaking. The protoplasts were harvested using centrifugation at 10,000 g for 3 min and resuspended in W5 solution (5 mM KCl, 125 mM CaCl_2_, 154 mM NaCl, and 31 mg/mL glucose) at a concentration of 1 × 10^6^ protoplasts/mL. After 30 min, the protoplasts were centrifuged at 15000 g for 4 min and resuspended in an equal volume of solution (0.4 M mannitol, 15 mM MgCl_2_, and 4 mM MES, pH 5.7) to allow the protoplasts to swell. For co-transformation, 75 μg target plasmids and 25 μg helper plasmid pHspNpt DNA were mixed with 1 mL protoplasts of *P. capsici*. For preparation of CK transformations, 25 μg of pHspNpt DNA was mixed with 1 mL protoplasts. The mixture was kept on ice for 5–10 min, and then 1.74 mL of 40% polyethylene glycol 4000 in 0.5 M CaCl_2_ and 0.8 M mannitol were added slowly. Subsequently, the suspension was gently mixed and placed on ice for 20 min, followed by addition of 10 mL pea broth containing 0.8 M mannitol. This mixture was then poured into a Petri dish that contained 10 mL pea broth with 50 μg/mL ampicillin and 0.8 M mannitol. After incubation for 14 h at 25°C, the mixture containing regenerated protoplasts was gently centrifuged at 12000 g for 5 min. The supernatant was removed, and the regenerated protoplast pellets were mixed with 10 mL pea broth agar (2%) containing 0.8 M mannitol and 30 μg/mL G418 (Sigma). Transformants appeared in the solid medium within 4 to 10 days at 25°C in dark conditions and were propagated in pea broth medium containing 30 μg/mL G418 (Sigma).

### Transcriptional analysis of target genes in silenced lines

To detect mRNA expression of 11 *PcNLP* targeted genes in the silenced lines, gene-specific primers of each *PcNLP* gene were designed; these are listed in Additional file 4: Table S1. The *β-Actin*, *β-Tubulin*, and *Ubc* (ubiquitin-conjugating enzyme) of *P. capsici*[69] were used as constitutively expressed endogenous controls and were used jointly as a reference to relate to the microarray data of the qRT-PCR detection. Each transformed line was first grown in 10% V8-juice liquid medium for three days at 25°C, and then total RNA was extracted from freeze-dried filtered mycelium based on the TRIZOL procedure (Invitrogen). Total RNA extractions of the different silenced lines and qRT-PCR were done as described above. WT is wild strain SD33; CK transformation is a strain expressing only the selected gene. SYBR green qRT-PCR assays were performed to determine individual *PcNLP* gene expression at the transcriptional levels. The expression levels of individual genes in SD33 or CK were assigned the value of 1.0 to allow comparison between lines. The threshold cycle (CT) values were determined automatically by instrument, and the fold changes of each gene were calculated by the equation 2^-△△CT^ according to a previous description [70]. Results were obtained from three repeated trials.

### Analysis of colony growth and sporangial morphology of silence transformants

For growth assays, the *P. capsici* strain SD33 (WT), CK (only the selected gene expression), and the silenced transformations were subcultured twice on 10% V8-juice agar medium. The colony radius of different strains was measured at 1, 3, 5, 7, 9 days of incubation.

To analyze sporangium production and zoospore release, strains of silenced transformations, SD33 and CK were individually inoculated into 20 mL sterile 10% V8 juice in Petri dishes. After four days incubation, the sporulating mycelia were washed with sterile distilled water at least three times, followed by incubation at 4°C for 1 h. The length and width of sporangia or/and number of zoospores were measured as described [29]. All tests were carried out in three replicates.

### Pepper leaf inoculation assay

For pepper leaf inoculation, strain SD33, CK transformations (positive control) and *PcNLP*-silenced lines were induced to produce zoospores as described above. Detached leaves of pepper at the fifth to sixth-leaf stages were placed in Petri dishes containing 1.5% (w/v) water agar. Each leaflet was spot-incubated with 2.5 μL of a zoospore suspension (1 × 10^5^ zoospores/mL) with each transformation, CK and SD33 strains, and then kept in darkness at 25°C. The leaves were inoculated with distilled water used as negative control. The length and width of the lesions were measured at 3 dpi. Mean lesion areas appearing on the pepper leaves inoculated with individual silenced strains were also calculated at 3 dpi. Bars represent the mean ± standard error of 14 leaves (*P* = 0.01 or *P* = 0.05). Pictures of the lesions were taken at 3 dpi, as most of the lesions were not intact at 5 dpi. The tests were repeated three times with 14 leaves in each experiment.

### Statistical analysis

Data were analyzed statistically using JMP Software (SAS Institute Inc., Cary, NC, USA). Data were subjected to one-way analysis of variance (ANOVA), and means were separated using Student’s multiple-range test (P = 0.05 or P = 0.01)

## Availability of supporting data

The data supporting the results of this article are included within the article.

## Competing interests

The authors declare that there are no competing interests.

## Authors’ contributions

XGZ carried out the design of the experiments and draft the manuscript. BAF carried out the molecular genetics, designed the gene silencing vector. XPZ participated in the statistical analysis and draft the manuscript. LF participated in the construction of the silencing vector. RFL participated in the qRT-PCR and gene silencing. DS participated in the *PcINF* gene clone and inoculation with pepper leaves. PT draft the manuscript and revised English for many times. All authors read and approved the final manuscript.

## Supplementary Material

Additional file 1: Figure S1 Sequence alignment of the 11 PcNLPs. The conserved cysteine is in box 1 or in box 2. The hepta-peptide motif ‘GHRHDWE’ is in box 3 and the C-terminal relatively conserved motif ‘QDLIMWDQ’ is in box 4. Arrowheads indicate potentially active sites. The signal peptide for each PcNLP is underlined. Blod indicates that the residues are conserved in all compared NLPs, whereas other colors denote sequences conserved only in some NLPs. The consensus line shows only those residues that are identical in 100% of the sequences. Click here for file

Additional file 2: Figure S3 The melting curve of each of the targeted *PcNLP* genes was amplified by qRT-PCR using specificity of the primers. Three housekeeping genes of *β-Actin*, *β-Tublin*, and *Ubc* were used as constitutively expressed endogenous controls and were used jointly as a reference to relate to the microarray data. The values of threshold cycle (CT) were ascertained automatically by instrument, and the fold changes of individual gene were calculated using the equation 2^-ΔΔCT^. The investigation was conducted twice, each with three independent biological replicates. Click here for file

Additional file 3: Figure S2 **A**: The complete reverse sequence of wild-type *PcNLP*1. Active site 112D was encoded by the nucleic acid (GTC) at site 430. Active site 120H was encoded by nucleic acid sequence (TGT) at site 408. Active site 123D was encoded by nucleic acid sequence (GTG) at site 400. Active site 125E was encoded by nucleic acid sequence (CTC) at site 390, indicated by underlining. **B**: The mutation of active site 112D→D112A in *PcNLP*1, the GAC was replaced by GCA at site 352, indicated by underlining. **C**: The mutation of active site 120H→H120A in *PcNLP*1, the CAC was replaced by GCC at site 464, indicated by underlining. **D**: The mutation of active site 123D→D123A in *PcNLP*1, the GAC was replaced by GCA at site 390, indicated by underlining. **E**: The mutation of active site 125E→E125A in *PcNLP*1, the GAG was replaced by GCA at site 480, indicated by underlining. **F**: The mutation of sitesD112/H120/D123/E125→D112A/H120A/D123A/E125A in *PcNLP*1. All the replaced bases are indicated by underlining. Click here for file

Additional file 4: Table S1 Primers used for RT-PCR and qRT-PCR. Click here for file

Additional file 5: Table S2 Primers used for PVX vector construction. Click here for file

Additional file 6: Table S3 Primers used for in vitro mutation of *PcNLP*1 potential active sites. Click here for file

Additional file 7: Table S4 Primers used for stable silence vector construction. Click here for file
