# Supplementary figures and images for: Characterization of necrosis-inducing NLP proteins in Phytophthora capsici
Source: BMC Plant Biol. 2014 May 8;14:126. doi: 10.1186/1471-2229-14-126 (PMC4023171; doi:10.1186/1471-2229-14-126)

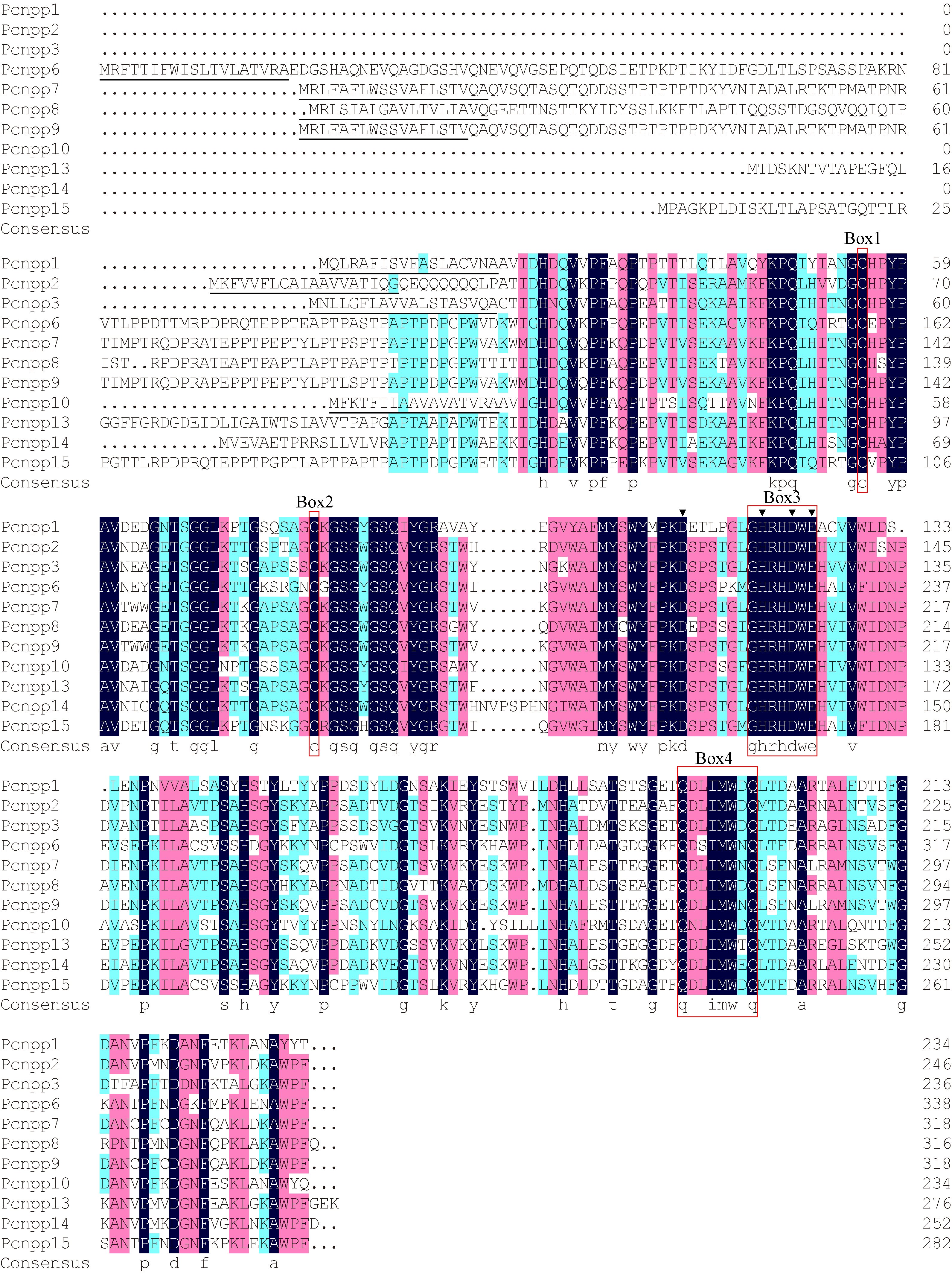

Supplement: Additional file 1: Figure S1 — Sequence alignment of the 11 PcNLPs. The conserved cysteine is in box 1 or in box 2. The hepta-peptide motif ‘GHRHDWE’ is in box 3 and the C-terminal relatively conserved motif ‘QDLIMWDQ’ is in box 4. Arrowheads indicate potentially active sites. The signal peptide for each PcNLP is underlined. Blod indicates that the residues are conserved in all compared NLPs, whereas other colors denote sequences conserved only in some NLPs. The consensus line shows only those residues that are identical in 100% of the sequences. [file 1471-2229-14-126-S1.jpeg]

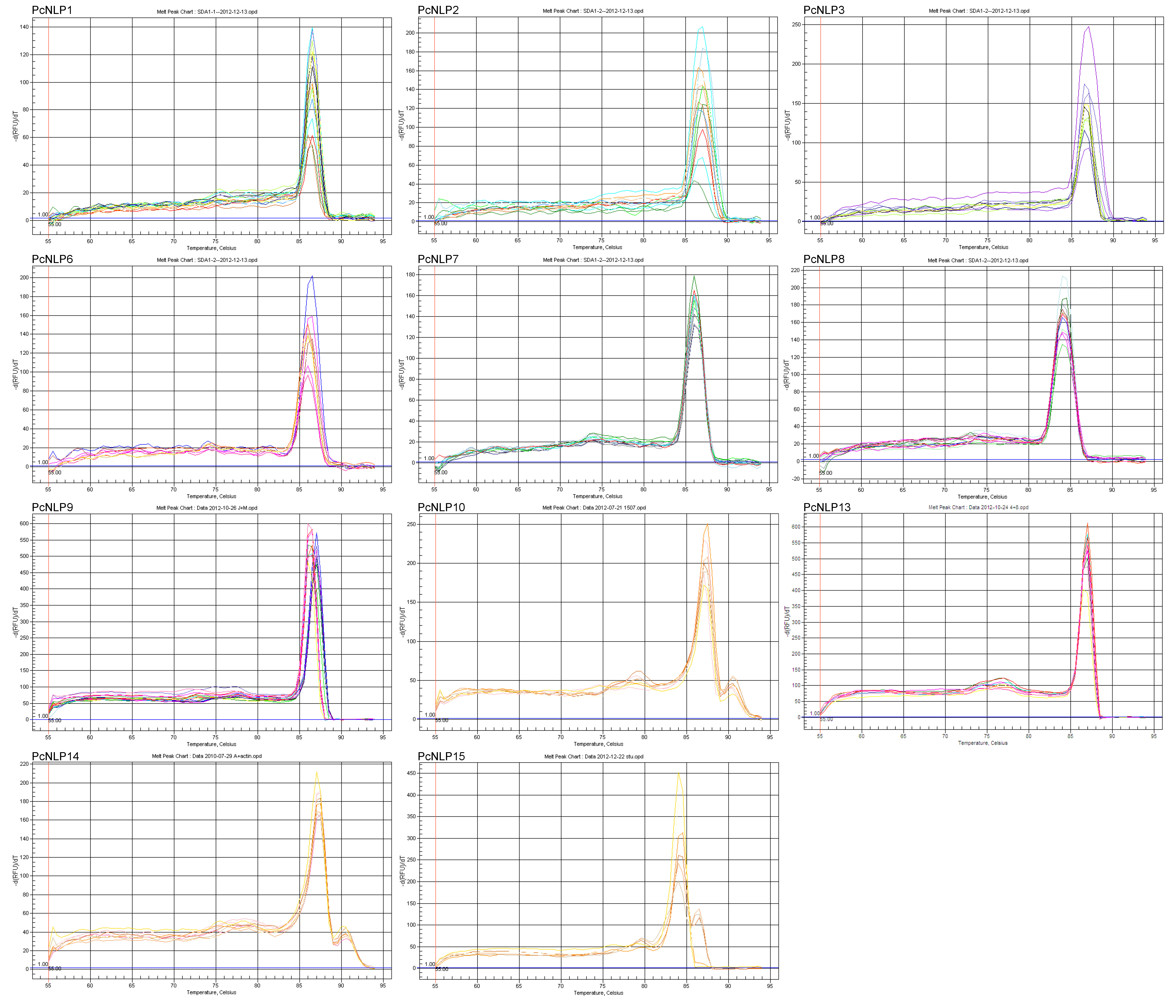

Supplement: Additional file 2: Figure S3 — The melting curve of each of the targeted PcNLP genes was amplified by qRT-PCR using specificity of the primers. Three housekeeping genes of β-Actin, β-Tublin, and Ubc were used as constitutively expressed endogenous controls and were used jointly as a reference to relate to the microarray data. The values of threshold cycle (CT) were ascertained automatically by instrument, and the fold changes of individual gene were calculated using the equation 2-ΔΔCT. The investigation was conducted twice, each with three independent biological replicates. [file 1471-2229-14-126-S2.jpeg]

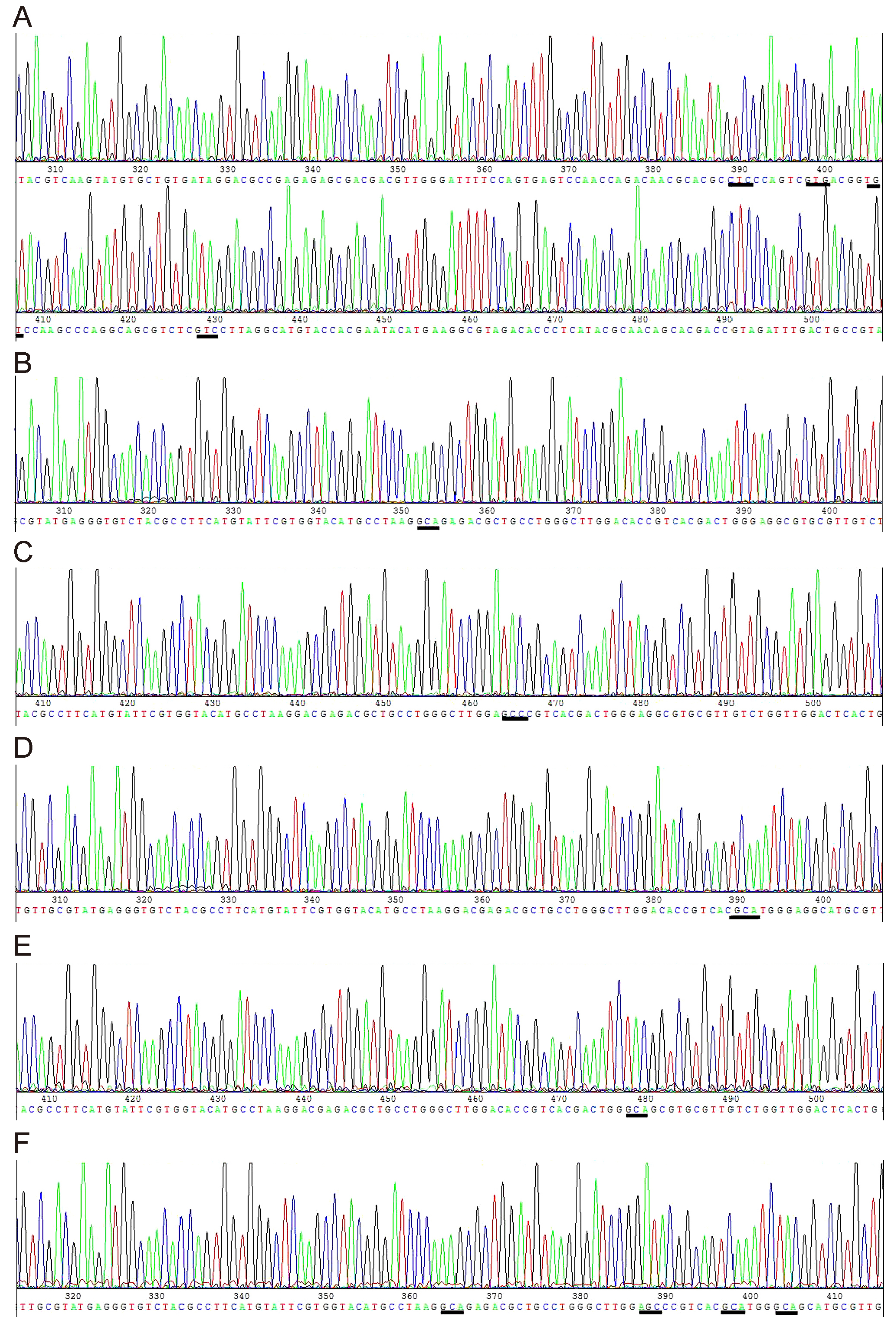

Supplement: Additional file 3: Figure S2 — A: The complete reverse sequence of wild-type PcNLP1. Active site 112D was encoded by the nucleic acid (GTC) at site 430. Active site 120H was encoded by nucleic acid sequence (TGT) at site 408. Active site 123D was encoded by nucleic acid sequence (GTG) at site 400. Active site 125E was encoded by nucleic acid sequence (CTC) at site 390, indicated by underlining. B: The mutation of active site 112D→D112A in PcNLP1, the GAC was replaced by GCA at site 352, indicated by underlining. C: The mutation of active site 120H→H120A in PcNLP1, the CAC was replaced by GCC at site 464, indicated by underlining. D: The mutation of active site 123D→D123A in PcNLP1, the GAC was replaced by GCA at site 390, indicated by underlining. E: The mutation of active site 125E→E125A in PcNLP1, the GAG was replaced by GCA at site 480, indicated by underlining. F: The mutation of sitesD112/H120/D123/E125→D112A/H120A/D123A/E125A in PcNLP1. All the replaced bases are indicated by underlining. [file 1471-2229-14-126-S3.jpeg]
