# Supplementary material for: Characterization of necrosis-inducing NLP proteins in Phytophthora capsici
Source: BMC Plant Biol. 2014 May 8;14:126. doi: 10.1186/1471-2229-14-126 (PMC4023171; doi:10.1186/1471-2229-14-126)
Supplement: Additional file 4: Table S1 — Primers used for RT-PCR and qRT-PCR. [file 1471-2229-14-126-S4.doc]

**Table S1** **Primers used for RT-PCR and qRT-PCR**

| **Genes** | **Primers** | **Nucleotide sequences (5`-3`)** | **Expected size** |
| --- | --- | --- | --- |
| *PcNLP*1 | *PcNLP*1F | GTTGGACTCACGGA AAATCC | 147 |
|  | *PcNLP*1R | GTGGTCAAGGATAACCCAACT |  |
| *PcNLP*2 | *PcNLP*2F | ATGAAATTCGTCGTTTTCCTC | 179 |
|  | *PcNLP*2R | AGTTGGGGTTTGAACTTCATC |  |
| *PcNLP*3 | *PcNLP*3F | TCCAAGCTGGAACTATCGAT | 166 |
|  | *PcNLP*3R | AGTCCACCACTGGTCTCTCC |  |
| *PcNLP*6 | *PcNLP*6F | ACCACCATCTTCTGGATCAG | 165 |
|  | *PcNLP*6R | AGAGTCTTGAGTCTGCGGTT |  |
| *PcNLP*7 | *PcNLP*7F | GACCCAAGATGACAGCAGTA | 196 |
|  | *PcNLP*7R | TAGGAGCTGGAGTAGGTGAC |  |
| *PcNLP*8 | *PcNLP*8F | GTTCGCAGGTACAACAAATT | 202 |
|  | *PcNLP*8R | AGATAGTGACAGGTTCAGGTTG |  |
| *PcNLP*9 | *PcNLP*9F | GACCCAAGATGACAGCAGTA | 180 |
|  | *PcNLP*9R | TGACAGGGTAGGCAAATATG |  |
| *PcNPP*10 | *PcNLP*10F | CTGAGCGATCATGTACTCCT | 171 |
|  | *PcNLP*10R | AGGATAGTAGACCGTGTAGCC |  |
| *PcNLP*13 | *PcNLP*13F | GTCGGCTCTTAGTGGTTATT | 156 |
|  | *PcNLP*13R | AGTCCACATGATGAGGTCTTG |  |
| *PcNLP*14 | *PcNLP*14F | GGAAGATCTTGGCTGTCA | 165 |
|  | *PcNLP*14R | GGTCCTGGTAGTCCCCTC |  |
| *PcNLP*15 | *PcNLP*15F | GCATGCTGGCTACAAGAA | 137 |
|  | *PcNLP*15R | AGGTCCTGAAAAGTCCCA |  |
| *β-Actin* of *P. capsici* | *β-Actin*F | ACTGCACGTTCCAGACGATC | 129 |
|  | *β-Actin*R | CCACCACCTTGATCTTCATG |  |
| *β-Actin* of pepper | *β-Actin*F | AGGGATGGGTCAAAAGGATGC | 290 |
|  | *β*-*Actin*R | GAGACAACACCGCCTGAATAGC |  |
| *β-Tublin* of *P. capsici* | *β-Tub*F | AGGAGATGTTCAAGCGTGTG | 129 |
|  | *β-Tub*R | GATCGTTCATGTTGGACTCG |  |
| *Ubc* of *P. capsici* | *Ubc*F | CGCCACTTAGAGCACGCTAG | 127 |
|  | *Ubc*R | CGCACAACAGTTACTAGGCAG |  |
