# Supplementary material for: Characterization of necrosis-inducing NLP proteins in Phytophthora capsici
Source: BMC Plant Biol. 2014 May 8;14:126. doi: 10.1186/1471-2229-14-126 (PMC4023171; doi:10.1186/1471-2229-14-126)
Supplement: Additional file 5: Table S2 — Primers used for PVX vector construction. [file 1471-2229-14-126-S5.doc]

**Table S2 Primers used for** PVX vector construction

| **Application** | **Primers** | **Nucleotide sequences (5`-3`)** |
| --- | --- | --- |
| *PcNLP*1 | *PcNLP*1F | *cccatcgat*GCTGTTATCGACCACGACCAGGTCGT |
|  | *PcNLP*1R | *cgaagcggccgc*TTAAGTGTAGTACGCGTTAGCTAGT |
| *PcNLP*2 | *PcNLP*2F | *cttagcggccgc*CAAGAGCAGCAGCAGCAACAACA |
|  | *PcNLP*2R | *acgcgtcgac*CTAGAAGGGCCAGGCCTTGTCCAG |
| *PcNLP*3 | *PcNLP*3F | *aaagcggccgc*GGAACTATCGATCACAACCAGGT |
|  | *PcNLP*3R | *acgcgtcgac*TTAGAATGGCCAAGCCTTACC |
| *PcNLP*6 | *PcNLP*6F | *tcccccggg*GAAGACGGTTCGCACGCTCAAAA |
|  | *PcNLP*6R | *acgcgtcgac*TTAAAACGGCCAGGCGTTTTCAA |
| *PcNLP*7 | *PcNLP*7F | *cccatcgat*CAAGTTTCTCAAACCGCTTCCCAGA |
|  | *PcNLP*7R | *tcccccggg*TTAGAACGGCCAAGCCTTGTCCAGT |
| *PcNLP*8 | *PcNLP*8F | *tcccccggg*GAAGAAACTACCAACTCAACGA |
|  | *PcNLP*8R | *acgcgtcgac*TCATTGAAAGGGCCAAGCTTTGG |
| *PcNLP*9 | *PcNLP*9F | *cccatcgat*CAAGTTTCTCAAACCGCTTCCCA |
|  | *PcNLP*9R | *tcccccggg*TTAGAACGGCCAAGCCTTGTCCA |
| *PcNLP*10 | *PcNLP*10F | *cccatcgat*GCAGTCATTGGCCACGACCAGGTC |
|  | *PcNLP*10R | *tcccccggg*CTACTGGTACCAGGCGTTCGCGAGC |
| *PcNLP*13 | *PcNLP*13F | *tcccccggg*ATGACCGACAGTAAAAACACCGTTA |
|  | *PcNLP*13R | *acgcgtcgac*CTATTTTTTTTCGCCAAATGGC |
| *PcNLP*14 | *PcNLP*14F | *cccatcgat*ATGGTGGAGGTGGCGGAGAC |
|  | *PcNLP*14R | *tcccccggg*TTAATCAAACGGCCAGGCCTTG |
| *PcNLP*15 | *PcNLP*15F | *cccatcgat*ATGCCAGCCGGCAAGCCCCT |
|  | *PcNLP*15R | *tcccccggg*TTAGAAGGGCCAAGCCTTCTCCAGTT |
| *PcINF*1 | *INF1*F | *cccatcgatat*GAACTTCCGTGCTCTGTTC |
|  | *INF1*R | *tcccccggg*TTACAGCGACGCGCACGTGTT |

All restrict enzyme sites are in italics.
