# Supplementary material for: Characterization of necrosis-inducing NLP proteins in Phytophthora capsici
Source: BMC Plant Biol. 2014 May 8;14:126. doi: 10.1186/1471-2229-14-126 (PMC4023171; doi:10.1186/1471-2229-14-126)
Supplement: Additional file 6: Table S3 — Primers used for in vitro mutation of PcNLP1 potential active sites. [file 1471-2229-14-126-S6.doc]

**Table S3 Primers used for in vitro mutation of *PcNLP*1 potential active sites**

| **Primers** | **Nucleotide sequences (5`-3`)** |
| --- | --- |
| NLP1F | CCATCGATGCTGTTATCGACCACG ACCAGGTCGT |
| NLP1R | GAAAGCGGCCGCAGTGTAGTAC GCGTTAGCTA GT |
| D112AF | AAGGCAGAGACGCTGCCTGGGCTTGGACACCGT |
| D112AR | ACGGTGTCCAAGCCCAGGCA GCGTCTCTGC CTT |
| H120AF | AAGGACGAGACGCTGCCTGGGCTTGGAGCCCGT |
| H120AR | ACGGGCTCCAAGCCCAGGCAGCGTCTCGTC CTT |
| D123AF | GGACACCGTCACGCATGGGAGGC |
| D123AR | GCCTCCCATGCGTGACGGTG TCC |
| E125AF | GGACACCGTCACGACTGGGCAGC |
| E125AR | GCTGCCCAGTCGTGACGGTG TCC |
| All F | AGGCAGAGACGCTGCCTGGGCTTGGAGCCCGTCACGCATGGGCAGC |
| All R | GCTGCCCATGCGTGACGGGCTCCAAGCCCAGGCAGCGTCT CTGCCT |
