# Supplementary material for: Characterization of necrosis-inducing NLP proteins in Phytophthora capsici
Source: BMC Plant Biol. 2014 May 8;14:126. doi: 10.1186/1471-2229-14-126 (PMC4023171; doi:10.1186/1471-2229-14-126)
Supplement: Additional file 7: Table S4 — Primers used for stable silence vector construction. [file 1471-2229-14-126-S7.doc]

Table S4 Primers used for stable silence vector construction

| **Genes** | **Primers** | **Nucleotide sequences (5`-3`)** |
| --- | --- | --- |
| *PcNLP*1 | *PcNLP*1F | *tcccccggg*ATGCATCCAA CGCGTTGGGA |
|  | *PcNLP*1R | *tcccccggg*GGCCCGACGT CGCATGC |
| *PcNLP*2 | *PcNLP*2F | *tcccccggg*ATGAAATTCG TCGTTTTCCT |
|  | *PcNLP*2R | *tcccccggg*CTAGAAGGGC CAGGCCTTG |
| *PcNLP*3 | *PcNLP*3F | *tcccccggg*ATGAACCTTC TGGGATTCCT |
|  | *PcNLP*3R | *tcccccggg*TTAGAATGGC CAAGCCTTACC |
| *PcNLP*6 | *PcNLP*6F | *tcccccggg*ATGAGGTTTA CCACCATCTT |
|  | *PcNLP*6R | *tcccccggg*TTAAAACGGC CAGGCGT |
| *PcNLP*7 | *PcNLP*7F | *tcccccggg*ATGAGGC TCTTCGCTTT |
|  | *PcNLP*7R | *tcccccggg*TTAGAACGGC CAAGCCTTGT |
| *PcNLP*8 | *PcNLP*8F | *tcccccggg*ATGAGGCTCA GTATCGCCTT |
|  | *PcNLP*8R | *tcccccggg*TCATTGAAAG GGCCAAGCTTT |
| *PcNLP*9 | *PcNLP*9F | *tcccccggg*ATGAGGCTCT TCGCTTTCCT |
|  | *PcNLP*9R | *tcccccggg*TTAGAACGGC CAAGCCT |
| *PcNLP*10 | *PcNLP*10F | *tcccccggg*ATGGAGCCCC TGAATATAAG |
|  | *PcNLP*10R | *tcccccggg* TTAGAAGGGC CAAGCCTTC |
| *PcNLP*13 | *PcNLP*13F | *tcccccggg*ATGACCGACAGTAAAAACACCGTTA |
|  | *PcNLP*13R | *tcccccggg*CTATTTTTTTTCGCCAAATGGC |
| *PcNLP*14 | *PcNLP*14F | *tcccccggg*ATGGTGGAGGTGGCGGAGAC |
|  | *PcNLP*14R | *tcccccggg*TTAATCAAACGGCCAGGCCTTG |
| *PcNLP*15 | *PcNLP*15F | *tcccccggg*ATGCCAGCCGGCAAGCCCCT |
|  | *PcNLP*15R | *tcccccggg*TTAGAAGGGCCAAGCCTTCTCCAGTT |

All *Sma*І restrict enzyme sites are in italics
